# Nanoparticle retention enables non-invasive detection of metastases by magnetic particle imaging in murine breast cancer models

**DOI:** 10.7150/thno.122259

**Published:** 2026-01-01

**Authors:** Preethi Korangath, Hayden Carlton, Toby Sanders, Olivia C Sehl, Suqi Ke, Abdul Rahman Mohtasebzadeh, Lyndsey Werhane, Cordula Grüttner, Chen Hu, Kathleen Gabrielson, Patrick W Goodwill, Robert Ivkov

**Affiliations:** 1 Dept of Radiation Oncology and Molecular Radiation Sciences, School of Medicine, Johns Hopkins University, Baltimore, Maryland, USA.; 2 Magnetic Insight Inc., Alameda, California, USA.; 3 Division of Quantitative Sciences, Sidney Kimmel Comprehensive Cancer Centre, School of Medicine, Johns Hopkins University, Baltimore, Maryland, USA.; 4 Micromod Partikeltechnologie GmbH, Rostock, Germany.; 5 Department of Molecular and Comparative Pathobiology, School of Medicine, Johns Hopkins University, Baltimore, Maryland, USA.; 6 Department of Mechanical Engineering, Whiting School of Engineering, Johns Hopkins University, Baltimore, Maryland, USA.; 7 Department of Oncology, Sidney Kimmel Comprehensive Cancer Centre, School of Medicine, Johns Hopkins University, Baltimore, Maryland, USA.; 8 Department of Materials Science and Engineering, Whiting School of Engineering, Johns Hopkins University, Baltimore, Maryland, USA.; 9 Institute for NanoBioTechnology, Whiting School of Engineering, Johns Hopkins University, Baltimore, Maryland, USA.

**Keywords:** iron oxide nanoparticles, macrophages, magnetic particle imaging, metastasis detection, stromal cells, tumor microenvironment

## Abstract

Early detection of metastatic disease improves cancer survival, yet existing modalities are limited in their detection capabilities. We propose that magnetic particle imaging (MPI), an emerging technology, can be used for early detection of primary tumors and metastases. MPI detects minute quantities of magnetic particles that act as “cold tracers” which accumulate in areas of high immune activity.

**Methods:** Pegylated Synomag® nanoparticles were intravenously injected into mouse models of breast cancer bearing primary tumors and spontaneously developed lung metastases. After 72 h, mice were subjected to three-dimensional MPI followed by structural imaging for co-registration. Non-tumor bearing mice served as controls for background signal correction and toxicity analysis. Animals were then sacrificed to collect tumors and organs of interest for two-dimensional MPI scans before fixing them for histopathological evaluation by hematoxylin and Eosin (H&E), Prussian blue, and immunohistochemistry staining. To further substantiate our findings towards clinical translation, tumor phantoms with nanoparticles were evaluated in a newly-built human scale MPI.

**Results:** Pegylated Synomag® nanoparticles showed a strong signal in both *in vitro* and *in vivo* models. Multiple macro and micro metastatic sites were identified by MPI and later confirmed by histology. *Ex vivo* quantitative analysis showed MPI can detect metastasis with high specificity and sensitivity, with positive correlations between tumor burden and macrophage population in the tumor microenvironment. Towards clinical translation, we also demonstrate nanoparticle detection in tumor phantoms using a human-scale MPI.

**Conclusion:** MPI using Pegylated Synomag® nanoparticles can successfully detect primary tumors and micrometastases away from large organs of the reticuloendothelial system. Nanoparticles were found in the tumor microenvironment, associated with stromal and immune cells, especially macrophages. This provides evidence to use MPI for noninvasive detection of highly inflammatory tumors and metastasis, as well as exploring their potential for other inflammatory diseases.

## Introduction

Cancer is the second leading cause of death worldwide, with metastatic disease dominating mortality 1, 2. The search for effective treatments continues, but evidence shows that when metastases are detected early and patients are prescribed timely treatments, outcomes improve 3. Yet, even with advanced imaging modalities such as X-ray computed tomography (CT), magnetic resonance imaging (MRI), and positron emission tomography (PET), early detection rates for many metastatic breast cancers remain low with little improvement in long-term patient survival 1-5. The limitation shared by clinical imaging modalities is that small or microscopic metastases fail to register in scans. This is especially true for microscopic lesions in bone or lungs because of limitations in size resolution and insufficient contrast with tissues displaying natural variations in intensity.

One of the main tracer imaging modalities used to detect metastatic lesions is PET, where an image is generated from the detection of radioactive material and often superimposed with anatomical scans, like CT or MRI. ^18^F-fluorodeoxyglucose (^18^F-FDG)/PET is a common tracer used to detect metastatic disease or recurrence 6. While clinically useful for cancer detection, PET comes with several limitations. From a patient safety standpoint, PET requires that the patient receive low dose radiation, which comes with inherent risks. Uptake of ^18^F-FDG is also non-specific to cancer cells, which can result in a signal originating from a variety of infections or other inflammatory diseases 7-9

Magnetic particle imaging (MPI) is a nascent, radioactively cold tracer imaging modality that measures a time dependent signal coming from the nonlinear response of magnetic nanoparticles to an oscillating magnetic field 10,11 and little or no signal from tissue. MPI detection sensitivity can be quite high for materials with ideal magnetic properties; several formulations of injectable magnetic nanoparticle suspensions with suitable MPI characteristics are readily available 12,13. Although still in preclinical research, MPI serves as a noninvasive method to quantify the ideal tracers accumulating in regions of interest; hence explored for its use in various biomedical applications like cell tracking, brain injury and others 14. Recent works have identified the utility of MPI to detect primary tumors with nanoparticles conjugated with targeting moieties 15-17. As opposed to ^18^F-FDG PET, where the tracer accumulates in areas of high glucose metabolism, iron oxide nanoparticle uptake has been found to associate strongly with the phagocytic immune cells present within the tumor microenvironment (TME), mainly macrophages 18,19. Tumor associated macrophages (TAMs) that actively promote tumor growth and invasion are associated with aggressive cancer types, high risk of metastasis and poor survival rate 20, 21. The preferential retention of nanoparticles in TME offers an opportunity to identify them with MPI. Yet, the main unanswered problem is whether enough of these particles can spatially accumulate in microscopic metastases to enable detection with MPI, particularly in locations that are challenging for other modalities, such as in the bones or lungs. Also, if detected, whether those tracers can be used in clinical scale scanners is still unexplored. Herein, we perform an imaging analysis on two different metastatic mouse models of breast cancer. We first performed *in vivo* MPI with co-registered anatomical imaging to determine the locations of any distant metastases. Afterwards, an *ex vivo* cutoff analysis of lungs with metastatic nodules correlated the scanner signal with overall tumor burden. Lastly, we assessed the clinical translation of our findings using a clinical-scale MPI head scanner.

## Materials and Methods

### Cancer cell line and reagents

Metastatic triple negative mouse mammary carcinoma cells transfected with luciferin, 4T1-luc were a generous gift from Dr. Saraswati Sukumar, Johns Hopkins University. These cells were grown in Roswell Park Memorial Institute (RPMI1640) media (Thermo Fisher Scientific Inc., Waltham, MA) supplemented with 10% FBS. Cells were authenticated using short tandem repeat (STR) analysis (data provided upon request) and matched against ATCC and Deutsche Sammlung von Mikroorganismen und Zellkulturen (DSMZ) databases to ensure their genetic origins. All other reagents used were of analytical grade.

### Nanoparticle synthesis and characterization

Hydroxyethyl starch coated pegylated Synomag®-PEG (SPEG) nanoparticles were obtained from Micromod Partikeltechnologie, GmbH, Rostock, Germany (Product code: 105-124-102). It was prepared from Synomag® Plain (SP) particles (Product-code: 105-00-102). SP were synthesized by a core shell method. The nanoflower-shaped core particles were prepared by a polyol method resulting in cores of a hydrodynamic diameter of about 50 nm 22, 23. Final coating with hydroxyethyl starch as shell material results in monodisperse SP particles with a hydrodynamic diameter of about 100 nm. SPs with a PEG 25.000-OMe surface (SPEG) were prepared by covalent binding of a MeO-PEG derivative to the starch surface of SPs.

Physical characteristics and measured analytical data provided by the manufacturer are given in Table S1. The hydrodynamic particle diameter, the size distribution and the zeta potential were measured with a Zetasizer Ultra (Malvern Panalytical). The particle size was measured at an iron concentration of 0.1 mg/ml in 0.22 µm filtered water. The zeta potential was measured at an iron concentration of 0.5 mg/ml in 1 mM KCl.

**Colloidal stability test:** The hydrodynamic diameter or Effective Particle Diameter (EPD), polydispersity index (PDI), and scattering count rate were assessed to test the colloidal stability of SPEG (70 µg/mL) nanoparticles in deionized water over a period of 96 h. For this, NanoBrook Omni (Brookhaven Instruments) was used.

**MPI Relaxometry:** Magnetic particle relaxometry was conducted using RELAX acquisition on the Momentum MPI scanner, using a drive field of 20 mT and a bias field of 160 mT. SPEG (7.5 µg) and Vivotrax (27.5 µg) nanoparticles were diluted in water to a volume of 20 µL then placed at the x = 0 position for relaxometry. The concentration of SPEG and Vivotrax was optimized to produce a peak signal = 1 a.u. Relax scans were performed in triplicate, then averaged to produce point spread functions for each nanoparticle.

**Transmission electron microscopy:** Nanoparticle samples (8 µL) were adsorbed to glow discharged (EMS GloQube) ultra-thin (UL) carbon coated 400 mesh copper grids (EMS CF400-Cu-UL), by floatation for 1 min followed by aspiration. Grids were imaged on a Hitachi 7600 TEM operating at 80 kV with an AMT XR80 CCD (8 megapixel).

### Mouse models

All animal studies were performed according to the NIH guidelines for the care and use of laboratory animals (Guide for the Care and Use of Laboratory Animals, 8th edition, National Research Council (US) Committee for the Update of the Guide for the Care and Use of Laboratory Animals. Washington (DC): National Academies Press (US); 2011). The Institutional Animal Care and Use Committee at Johns Hopkins University approved all animal studies under protocol number M024M16. We maintain a colony of transgenic human HER2 overexpressing transgenic mice (huHER2) in the FVB/NJ background that spontaneously develop mammary tumors that metastasize to the lungs 24. These mice were originally obtained from Genentech (San Francisco, CA) under a Materials Transfer Agreement. FVB/NJ and BALB/c (8 weeks old female) mice were purchased from Jackson Labs, Bar Harbor, ME. All mice were fed a normal diet and water *ad libitum*. They were maintained in the normal 12 h of light and dark. All animals were monitored closely for any distress or pain throughout the study period.

### *In vivo* study design

4T1-luc model: Cells (0.025x10^6^ in 50µL PBS) were orthotopically injected to 4th mammary gland of 8 weeks old BALB/c mice under isoflurane anesthesia. Primary tumor growth was monitored by measuring tumor volume twice weekly. On the 4th week, bioluminescence imaging was performed in the *In Vivo* Imaging System (IVIS Spectrum Imager - PerkinElmer, Waltham, MA) after intraperitoneal injection of D-luciferin (Catalog #88291 - Thermo Fisher Scientific Inc., Waltham, MA). When bioluminescence was detected around the lung area, mice (n = 10) received intravenous injection of SPEG (30 mg/kg b.wt or 0.6mg Fe/mouse) nanoparticles and were imaged as described below. Another cohort of 4 mice with confirmed lung bioluminescence served as PBS injected controls of which only one underwent 3D MPI scanning.

HuHER2 transgenic mice with tumors: These transgenic mice start to develop palpable mammary tumors when they are around 7 months old. They develop single or multiple mammary tumors over time. We monitor and measure all mammary tumors until they are around 1500-2000 mm³ in total volume. These mice were randomly assigned to one of two groups (n = 5-11/group) to receive a single intravenous injection of PBS or SPEG (30 mg/kg b.wt or 1mg Fe/mouse). Three out of 5 mice in the PBS cohort and 4 out of 11 in the SPEG cohort underwent 3D MPI scanning. The rest were evaluated by 2D MPI only.

HuHER2 transgenic mice without tumors: Age-matched huHER2 transgenic mice without any palpable tumors (only 77% of mice develop mammary tumors in this model) were used as control cohorts as older mice tend to have increased inflammation in lungs. These mice were injected with SPEG (1mg Fe/mouse - n = 3/group) and imaged in the Momentum scanner.

FVB/NJ and BALB/C wild type normal mice: Young female FVB/NJ or BALB/C mice (8-10 weeks old) were also used in this study as control cohorts with no tumor and hence no tumor associated inflammation in any organs. We injected SPEG (0.6 mgFe (BALB/c) or 1 mgFe (FVB/NJ) Fe/mouse - n = 3-4/group) to these mice and performed imaging as described below.

A separate cohort of BALB/C wild type normal mice and age-matched older huHER2 transgenic mice was used for the toxicity study (n = 8/cohort). Mice received PBS or SPEG through the tail vein (0.6 mgFe (BALB/c) or 1 mgFe (FVB/NJ) Fe/mouse - n = 4/group). After 72 h, all mice were sacrificed to collect blood from the heart for serum biochemical analysis. Liver function and kidney function parameters, along with inflammation marker C-reactive protein, were assessed in all samples using custom panels through IDEXX BioAnalytics (North Grafton, MA) service.

### *In vivo* magnetic particle imaging and micro-computerized tomography

Magnetic particle imaging (MPI) scans were done using a Momentum® scanner (Magnetic Insight, Alameda, CA). After 72 h of nanoparticle or PBS injection, the mice were anesthetized in a 2-3% isoflurane/oxygen chamber (Matrx VIP 3000, Midmark, Dayton, OH). The time point of 72 h was selected based on prior studies and our unpublished data that showed complete clearance of nanoparticles after 72 h from circulation 25,26. They were then positioned and secured in the holder attached to the scanner with a continuous flow of anesthesia. Body temperature was maintained between 34-36° C with the aid of circulating water tubing attached to the holder and monitored using a rectal temperature probe (Fiber optic 1 mm sensor, Small Animal Instruments, Stony Brook, NY). Similarly, respiration was also monitored using a respiratory sensor secured on the mouse's lung area, connected to PC-SAM monitor software (Small Animal Instruments, Stony Brook, NY). The respiratory rate was kept between 30 to 40 breaths/min by controlling the isoflurane flow rate. Five fiducial markers were placed at arbitrary locations along the holder; the fiducials consisted of ~1 µL aliquots of Vivotrax® nanoparticles in micro centrifuge tubes at a concentration of 5.5 mgFe/mL; the concentration was high enough to distinguish the markers on both MPI and X-ray imaging.

The mice first underwent 3D isotropic MPI scans in the “Standard” scanning configuration, with a gradient strength and RF excitation field strength of 5.7 T/m and 5 mT, respectively. A total of 21 radial slices were measured for each mouse within a 6 cm x 12 cm area that encompassed the entire body. Following 3D scanning, the mice were transported (still fixed in the holder) to an IVIS SpectrumCT *In Vivo* Imaging System (Perkin-Elmer, Shelton, CT, USA) for micro-computerized tomography (µCT) imaging. Each scan (50 kV at 1 mA) was 720 projections with a 20 ms exposure time. Both the MPI and µCT scans were then converted to .dcm image stacks and viewed using an MPI-specific viewing software, MagImage^®^. Using the fiducial markers as a guide, the anatomical and MP images were co-registered; each tumor, as well as the lungs, was segmented and the total summed signal from every voxel was recorded as the quantity “MPI signal”.

### 3D image reconstruction

The 3D image reconstruction was evaluated using a new 3D model-based approach, which is a 3D FFL projection model generalization of the 2D FFL model introduced in 27. The 2D FFL model accounts for all parameters in the scans, such as gradient strength, drive amplitude, FFL trajectory, and nanoparticle size. To generalize the 2D model, for memory purposes, the raw signal data is first compressed into its harmonic bands at harmonics 2 through 5, each at 1 kHz of total bandwidth. Then the basic 2D MPI model for each scan angle is the same as that in 27, but in this new compressed data format with corresponding model changes. The 3D MPI model is completed by combining a CT projection operator with the 2D models for all angles. The image reconstruction was then accomplished by iteratively solving a maximum a posteriori (MAP) estimator constructed with the 3D MPI model. A total of 250 iterations were used for each data set.

### 3D image analysis

After co-registering the reconstructed images with µCT, we quantitatively compared the intratumor MPI signal with that of normal muscle. For each tumored mouse, we segmented a small (~35 mm^3^) spherical volume of the tumor along the periphery, which was typically the most signal intense area. Additionally, we segmented a spherical region of identical volume in the lower left leg muscle. We recorded the average MPI signal within each of these volumes for each mouse.

### Euthanasia

After live imaging with MPI and µCT, all mice were euthanized to collect blood (in EDTA-coated tubes) and tissues of interest. This includes tumors, lung, liver, spleen, inguinal lymph nodes, sternum (for bone marrow), kidneys and intestine. The lung was collected after injecting 2 mL of PBS through the trachea to ensure expansion of the lungs after fixation for better visualization of tumor nodules in the lungs. In some cases, brain, bone tissue and other areas of bright spots detected by MPI were also collected. All tissues and 100 µL of blood were then imaged in the scanner. After imaging, the whole lung and a piece of tissue from all other organs were fixed in 10% formalin and processed for histology and immunohistochemistry (IHC) as described below.

### *Ex vivo* magnetic particle imaging of organs

To correlate the MPI signal with iron content, a mass/concentration gradient was prepared for SPEG which consisted of 9 samples (0, 0.0005, 0.001, 0.005, 0.01, 0.05, 0.1, 0.5 and 1 mg Fe) with increasing iron content between 0 and 1000 µg (injected mass). The gradients were imaged as a 2D projection scan in “Standard” mode with a gradient field of 5.7 T/m and an RF excitation field of 10 mT. After scanning, the gradient samples for SPEG were segmented using MagImage® and the total MPI signal was recorded. The MPI signal from each sample was plotted against the total iron mass within each sample and the resulting graph was fit using linear regression. After euthanasia, we removed and imaged each organ to quantify nanoparticle accumulation. The scans used the same parameters described for the nanoparticle calibration (Standard, 5.7 T/m, 10 mT). A higher RF excitation field was used for the *ex vivo* analysis to amplify the (often small) signal from the nanoparticles within each organ. After imaging, individual organ scans were converted to a single .dcm file and imported into MagImage®. The total scan area was kept constant at 6 cm x 6 cm, or 241 pixels x 241 pixels, for a total of 58,081 pixels per scan. The entire scanned area was segmented and, similar to the 3D *in vivo* scans and calibration, the summed scalar pixel values within the scan area were recorded as “MPI signal”. Additionally, we calculated the signal to noise ratio (SNR) for the organs with nanoparticles present using the following equation: *SNR = (S_NP_ - S_PBS_)/SD_PBS_*, where *S_NP_* is the mean scalar pixel value from the organ after nanoparticle injection and *S_PBS_* / *SD_PBS_* is the mean/standard deviation scalar pixel value from the corresponding organ after PBS injection 28.

### Histology

Tissues fixed in 10% formalin for 48 h were processed and embedded in paraffin blocks. Through Johns Hopkins core facility, tissues were sectioned and stained with hematoxylin and Eosin (H&E) for visualizing cell morphology. Four serial sections (100 µm apart) were stained with H&E to detect tumor nodules in the lung from 4T1-luc tumor bearing mice. Prussian blue (PB) staining was performed to visualize iron nanoparticle distribution 29. Charged unstained slides were used for immunohistochemistry (IHC). HER2 - IHC was used to determine metastatic nodules in huHER2 mice. IHC for immune and stromal cell markers was performed on huHER2 tumors and lungs collected from PBS and SPEG treated mice to determine their spatial distribution and correlate with that of nanoparticles from Prussian blue staining. For tissue toxicity analysis, liver, kidney, lungs, spleen, intestine, bone marrow and lymph nodes sections were stained with H&E. From each cohort of SPEG injected BALB/c and HuHER2 transgenic mice, 5 randomly selected mice tissues were analyzed by board certified veterinary pathologist (K.G) under a microscope.

### Immunohistochemistry

Immunohistochemistry for human HER2 and mouse F4/80 was performed on lung sections to identify HER2+ve metastatic tumor nodules and macrophage distribution respectively in the huHER2 transgenic cohort. F4/80 staining was also conducted on lung sections from 4T1-luc mice, old huHER2 transgenic mice without tumors, FVB/NJ and BALB/C wild type mice.

For HER2 IHC of the lung tissue, five serial sections of 100 µm apart were used to determine the number and area of lung metastatic nodules in each mouse. The tissues were sectioned on charged slides, antigen retrieval was performed in a steamer for 45 min with 10 mM citrate buffer after deparaffinization on a slide warmer, followed by xylene and serial washing in 100%, 95%, and 70% ethanol. Slides were then dipped for 5 min in PBS containing Tween, followed by blocking with endogenous peroxidase blocking solution (Agilent, Savage, MD) for 5 min. After washing with Tris-buffered saline with Tween (TBS-T), sections were incubated with anti-human HER2 antibody (29D8 - 1:400; Cell Signaling Technology, Danvers, MA) for 45 min at room temperature. After washing in TBS-T, sections were incubated with secondary antibody (PowerVision Poly-HRP anti-Rabbit IHC Detection Systems, Novocastra, Leica Biosystems, Buffalo Grove, IL) for 30 min at room temperature. The slides were further washed in TBS-T and developed with DAB fast (MilliporeSigma, St. Louis, MO) for 20 min at room temperature and counterstained with Mayer's hematoxylin (Lillie's modification) (Agilent, Savage, MD). F4/80 immunostaining was performed at the Oncology Tissue Services Core of Johns Hopkins University using a Ventana Discovery Ultra autostainer (Roche Diagnostics). Briefly, following dewaxing and rehydration on board, F4/80 epitope retrieval was performed using target antigen retrieval buffer (Dako/Agilent - S170084-2 Santa Clara, CA) at 96 °C for 48 min. Anti-F4/80 antibody (1:2000 dilution; Biorad MCA497, Hercules, CA) was applied for 60 min at 36 °C. Slides were then incubated with rabbit anti-rat linker antibody (1:500 - Vector Labs AI4001, Newark, CA) at 36 ºC for 32 min. Linker antibodies were detected using an anti-rabbit HQ detection system (catalog# 7017936001 and 7017812001, Roche Diagnostics, Indianapolis, IN). This was followed by incubation with Chromomap DAB IHC detection kit (catalog # 5266645001, Roche Diagnostics), counterstaining with Mayer's hematoxylin, dehydration and mounting.

For determining pattern of distribution, huHER2 tumors were stained for endothelial cells using CD31 (1:40 - Dianova- DIA 310, Hamburg, Germany), tumor cells using HER2 (1:400; Cell Signaling Technology- 29D8 Danvers, MA), macrophages using F4/80 (1:2000 - Serotec, now Biorad MCA497, Hercules, CA), fibroblast using alpha- smooth muscle actin (a-SMA -1:200 - Abcam ab5694, Waltham, MA), dendritic cells using CD11c (1:100 - Cell Signaling 97585S, Danvers, MA), collagen using Masson's trichrome and nanoparticles by Prussian blue (performed at JHU core facility). A detailed IHC procedure using all these antibodies is described elsewhere 19.

### Histology image analysis

All slides were digitized using the NanoZoomer-SQ digital slide scanner (Hamamatsu Photonics, Shizuoka, Japan) and evaluated in Aperio Imagescope software (version 12.4.6, Leica Biosystems, Deer Park, IL). For calculating tumor area in lungs, HER2+ve tumor areas identified from IHC slides of huHER2 mice or tumor area confirmed from H&E slides by Pathologist (Dr. Gabrielson), were manually annotated using the pen tool. A few mice had weakly positive adenomas of mouse origin, and those were also included in the analysis. The software then calculates the number of nodules and the area of each annotated region using the built-in algorithm. This can be accessed through the View/Annotations tab in the software. For F4/80+ve cell number analysis, QuPath software v0.5.0 (GitHub, San Francisco, CA) was used. Randomly selected 20 equal areas in each slide were subjected to a fast cell count algorithm in the software using the following settings:

Cell detection channel: DAB

Gaussian sigma: 1.5

Background radius: 15

Uncheck - Use difference of Gaussians

Cell detection threshold:0.25

DAB threshold: 0.1

Detection object diameter: 100

### Nanoparticle detection in a clinical-scale scanner

To create an anatomically relevant tumor phantom, an MRI of the brain of a patient with glioblastoma was obtained from Cancer Image Archive 30, 31. The tumor volume was segmented from MRI in 3DSlicer, then used to generate a fillable hollow tumor phantom and another tumor phantom with a 3-mm wide fillable tumor rim. The tumor phantoms were 3D printed with clear resin (Formlabs v4.1). SPEG was loaded at a concentration of 0.3 mg (25 µg/mL) and 2.55 mg (212.5 mg/mL) in the hollow tumor phantom, representing weakly and strongly enhancing tumors. SPEG was loaded at 2.55 mg (212.5 mg/mL) in the rim tumor phantom. Phantoms were imaged individually within a 22 x 22 x 20 cm field of view in a human head-sized MPI scanner (Magnetic Insight Inc.) for an acquisition time of 10 min. The clinical-scale MPI device has a 60 cm magnet-free bore and produces images using a field free point with 0.28 x 0.28 x 0.55 T/m gradient strength.

### Iron uptake by macrophages and MPI

Iron uptake by cultured macrophages of distinct phenotype assay was carried out as described before 18. Briefly, RAW264.7 cells (ATCC, Manassas, VA) maintained in DMEM with 10% heat-inactivated FBS were treated with LPS (100 ng/ml; Sigma-Aldrich, St. Louis, MO) and IFN-γ (50 ng/ml; Miltenyi Biotech, Germany) for 24 h to get the M1 macrophage phenotype. To induce cells into the M2 phenotype they were treated with IL-4 (10 ng/ml; Miltenyi Biotech, Germany) for 24 h. Cells grown in complete media alone served as an uninduced M0 phenotype. Induced and uninduced cells (1 million) were then incubated with SPEG nanoparticles (0.5 mg/ml) for 24 h. After incubation, cells were washed thoroughly with PBS four times and processed for intracellular iron content analysis by ferene-s assay 32. Experiments were independently repeated five times. Cell pellets of 14 x 10^6^ M1- and M2- induced macrophages were fixed in 10% formalin, then imaged with human-scale MPI with image dimensions 22 x 22 x 10 cm (n = 3).

### Dynamic range simulations and shine through phantom experiment

Simulations were performed to test the potential dynamic range limitations within MPI from a theoretical point of view, assuming a noise free system and ideal acquisition data. Dynamic range is defined as the ratio of two nanoparticle sources, which ranged from 10^1^ to 10^3^. For these simulations, synthetic data were generated by modeling a Momentum® system with a 5.7 T/m gradient, acquiring x and z collinear scans at 10 mT drive amplitudes, and using 2D projection imaging only. An ideal phantom containing superparamagnetic nanoparticles was simulated in the scanner, which contained two-point sources at different intensities (Fe concentrations). The magnetic properties were modeled assuming ideal spherical particles with no relaxation effects, and with the magnetic saturation properties observed for Synomag®. The time domain received signals were then generated by implementing the MPI signal modeling equations given the setup just described (see equation (5) in 33). The reconstructions were performed by implementing a model-based reconstruction using the simulated time domain signals 27. The two sources were set at particular distances apart from one another, moving further out until the small intensity point source was disjointly detected from the large intensity source in the reconstructed image at the expected location.

These simulations were validated through phantom experiments. The shine-through phantom consists of a central well and 6 satellite wells. Each well is 1.5 mm in diameter, and each satellite well was separated from the central well at an incremental distance of 2.5 mm so that the most distant well is 20mm away from the center. The central well was then loaded with 300 µg of SPEG nanoparticles and each satellite well with 30 µg of SPEG, which recapitulates an average difference in total measured iron in liver and metastatic lungs in the 4T1-luc model. Phantoms were then scanned on MPI using the same parameters we used for 3D mouse imaging and analyzed as described above.

### Statistical and diagnostic threshold analysis

To evaluate whether the MPI signal could distinguish between mice with and without lung metastasis, we performed a classification threshold analysis based on the Youden index, defined as Sensitivity+Specificity-1. The threshold that maximized this index was identified using the full dataset. Analyses were conducted separately for two experimental cohorts, as well as for a combined dataset that pooled both. All threshold analyses were conducted using the cutpoint package in R (version 4.4.1). To assess the robustness of the identified thresholds and the variability in diagnostic performance, we performed 1,000 bootstrap resampling iterations with replacement. For each iteration, the threshold, sensitivity, specificity, positive predictive value (PPV), and negative predictive value (NPV) were calculated. We report diagnostic metrics computed from the full dataset, as well as the frequency with which each threshold was selected across bootstrap samples. Ninety-five percent confidence intervals for sensitivity, specificity, PPV, and NPV were derived using the percentile method based on the bootstrap distribution.

Additional statistical analyses were performed using standard non-parametric methods. Spearman's correlation coefficients were calculated to evaluate the relationship between the raw MPI signal and tumor weight, the normalized MPI signal and area of lung metastases, and the normalized MPI signal and F4/80 expression. Group comparisons between cohorts shown in Figures 2, 3 and 5 were conducted using Graphpad Prism (Mann-Whitney). All statistical tests were two-sided, and p-values less than 0.05 were considered statistically significant.

## Results and Discussion

### MPI detects primary and metastatic tumors

Metastatic disease can occur among all breast cancer subtypes, but triple-negative and HER2-positive breast cancers are two that frequently present aggressive and invasive features leading to higher rates of distant and progressive disease 34, 35. Few murine models spontaneously develop metastases or progressive metastatic disease that recapitulates the complex biology observed in humans. Immune competent mice are necessary to study the interaction between the host immune system and the pharmacokinetics of nanoparticles *in vivo* 36, 37. Following this rationale, we selected two different mouse models of breast cancer: a syngeneic (BALB/c) 4T1 cell line (triple-negative breast cancer (TNBC)) transfected with firefly luciferase gene (4T1-luc) 38, 39; and a genetically-engineered mouse model (GEMM) of human HER2 overexpressing breast cancer (HER2+ breast cancer) 24. Both models are immunocompetent and develop distant metastases in the lungs and other organs. In many ways, these models mimic essential features of human disease, including unpredictable progression to metastatic disease; these properties make them ideal models for testing with MPI.

A dense “nanoflower” iron oxide core of aggregated iron oxide crystallite nanoparticles, Synomag^®^ 22 was selected due to prior analysis of image quality and thermal output of this commercially available magnetic colloid 40-45. The Synomag^®^ nanoparticles were coated with polyethylene glycol (PEG), which we denoted as SPEG. Coating nanoparticles with PEG increases nanoparticle extravasation into the TME by reducing sequestration of nanoparticles by liver-resident macrophages, thereby increasing blood circulation time 46. Our *in vitro* characterization of SPEG shown in Figure S1A-C demonstrates that the core size of SPEG is consistent with uncoated Synomag^®^ (transmission electron microscopy), that SPEG has superior MPI signal and resolution characteristics than Vivotrax (magnetic relaxometry) and is stable in solution over 96 hours (dynamic light scattering). Using a cohort of normal BALB/c mice and huHER2 GEMMs without tumors, we performed toxicity analysis of SPEG nanoparticles at the same doses and found no difference in any of the parameters analyzed like liver function, kidney function or inflammatory marker, C-reactive protein (CRP), when compared to PBS injected mice (Figure S1D-E).

**Figure 1:**
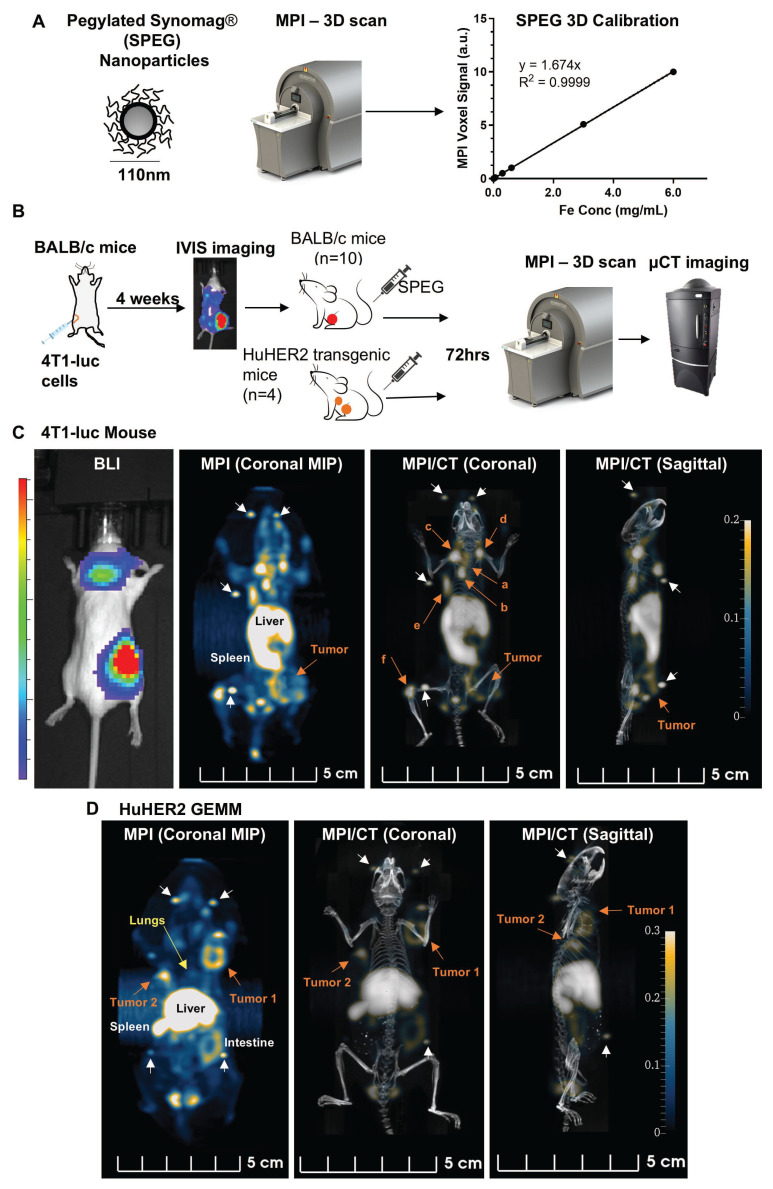
** MPI detects tumors and metastasis in breast cancer models.** (A) A pegylated Synomag nanoparticle (SPEG) construct was calibrated by measuring the MPI signal at different concentrations (0, 0.003, 0.006, 0.03, 0.06, 0.3, 0.6, 3 and 6 mgFe/mL). A dose dependent linear increase in signal with iron concentration was noted. (B) Schema of the study design (see methods for details). Mice (n = 10) with confirmed metastases with bioluminescence (BLI) were intravenously injected with SPEG nanoparticles (0.6 mgFe/mouse). HuHER2 transgenic mice (n = 4) with spontaneous mammary tumors (~1800 mm^3^) were also injected with SPEG nanoparticles (1 mgFe /mouse). (C) Each 4T1-luc tumor bearing mouse subjected to whole body 3D MPI scanning 72 h post injection showed MPI signal in the primary tumor (orange arrow) and distant metastatic areas including (a) lungs, (b) chest cavity (c-e) lymph node adjacent to right and left 1st and 2nd mammary gland, and (f) left leg (orange arrows). Fiducials are marked with white arrows. Representative image of 4T1-luc mouse 77 shows the 3D MPI scan both as a maximum intensity projection (MIP) and co-registered with microCT. (D) MPI MIPs and 3D scans of SPEG injected huHER2 transgenic mouse 192 are shown, which highlight the primary tumors (orange arrow) and metastatic nodules in the lungs (yellow arrow).

As shown in Figure 1A, we verified the linear relationship between the MPI signal and SPEG nanoparticles. We then injected SPEG nanoparticles intravenously into 4T1-bearing BALB/c mice and GEMMs bearing HER2+ tumors. Seventy-two hours after injection, whole-body 3D MPI was performed with micro-computed tomography (µCT) scanning for anatomical co-registration (Figure 1B). After the scans were acquired, 3D MPI reconstruction was performed using a 3D generalization of the methodology introduced in 27, which significantly improved the resolution of the images. Tumor bearing mice that received phosphate buffered saline (PBS) injection served as negative controls (Figure S2A-B). Additionally, we injected SPEG into normal BALB/c mice, FVB/NJ mice, and age-matched huHER2 GEMMs without tumors to compare background signal (Figure S2C-E).

The MPI scans of the 4T1-luc mice showed an intense signal in several key regions in the representative mouse: the tumor area, lungs, chest cavity, lymph node adjacent mammary glands (right 1^st^ and left 2^nd^), and left leg (Figure 1C, Movie S1). Maximum intensity projections (MIPs) of 3D MPI and MPI co-registered with µCT are shown. These regions become more apparent when compared to control PBS-injected tumored or SPEG-injected wild type mice (Figure S2). Quantitative analysis of primary tumor area (35 mm^3^) with a similar area in the leg muscle region showed significantly higher MPI signal in tumors (Figure S2F). The highest MPI signal corresponded to the location of the liver and spleen. This was expected, as these organs are filled with phagocytic cells, especially macrophages, which engulf nanoparticles 47. Bioluminescent Imaging (BLI) of 4T1-luc tumors showed luc+ cells at the location of the primary tumor in the right inguinal mammary gland and at sites of suspected metastases in distant regions, particularly in the chest cavity (Figure S3); whereas MPI showed additional areas of intense MPI signal (Figure 1C, Figure S3). Following SPEG injection in the huHER2 mice, MPI signal was detected in regions with palpable tumors and the lungs (Figure 1D, Figure S2G, Movie S2). No MPI signal was detected in any mice injected with PBS, thus demonstrating a low background signal in the absence of a magnetic tracer (Figure S2B, Movie S3). Similar to 4T1-luc tumor-bearing mice, the liver, spleen and lymph nodes of huHER2 mice displayed high MPI signal. In normal BALB/c, FVB/NJ, and huHER2 transgenic mice without tumors, we saw nanoparticle accumulation predominantly in organs like liver, spleen and lymph nodes, which all have a substantial macrophage population (Figure S2C-D and Movie S4-6), creating MPI signal background.

The relatively high MPI signal from primary tumors was confirmed by BLI, gross pathology examination, and/or palpation; however, suspicious signals from other regions required additional microscopic analysis. Histopathology serves as the gold standard for cancer diagnosis and is recommended for the rigor and reproducibility of translation studies 48, 49. Suspicious lesions identified by MPI were assessed by hematoxylin & Eosin (H&E) staining. For all lesions identified by 3D *in vivo* MPI, histopathology confirmed the presence of tumors (Figure S4) for the mouse shown in Figure 1C. In all 4T1-luc tumor-bearing mice injected with SPEG (n = 10), we confirmed the presence of multiple metastatic nodules in the lungs (Figure S5) and in other areas with high MPI signal (Figure S6, S7). The presence of nanoparticles in these nodules was confirmed by Prussian blue staining (Figure S8). Except for the primary tumors and lungs, both BLI and µCT failed to register these other sites as potential areas bearing small metastases. In one out of ten mice, there was a high MPI signal in the head area. This mouse developed a subcutaneous metastasis adjacent to the salivary gland, which projected next to the left eye which we confirmed by histology. Co-registration with µCT confirmed the exact position of the MPI signal (Figure S9) and Prussian blue analysis showed nanoparticle presence in the inflamed salivary gland adjacent to the metastatic nodule. The sensitivity of MPI for micrometastases detection is expected to vary depending on tracer dose, imaging timepoints, and biological variability in metastatic progression (as shown in Figure S3). We noted most of the distant micrometastases detected by MPI in the mouse shown in Figure 1C were undetectable by BLI or CT (Figure S10A). Taken together, the data demonstrate that MPI with SPEG nanoparticles can detect primary and metastatic lesions in mice, including micrometastases, that were undetectable by BLI. Quantitative analysis of tumor MPI signal compared to equal area on leg muscle, like PET image analysis 50, showed statistically significant MPI signal in primary tumors as expected (Figure S2F-G). Although qualitative visualization enabled the detection and later confirmation of metastases in multiple areas accurately by histology, quantitative analysis of 3D lungs was obscured by the high signal interference from the liver (Figure S10B). It has been reported that in clinical practice, qualitative analysis of PET images is widely used for detection and quantitation is useful and necessary for determining therapy effects 51-54.

In huHER2 GEMMs, normally about 50% of primary tumor bearing mice will develop lung metastasis and this can be confirmed only after histological analysis of lung tissue. We detected MPI signal in the lung area for half of tumor bearing mice injected with SPEG (Figure S11), which warranted further histological investigation as described below.

### MPI of the excised lung enables lung metastasis detection with high specificity and sensitivity

**Figure 2:**
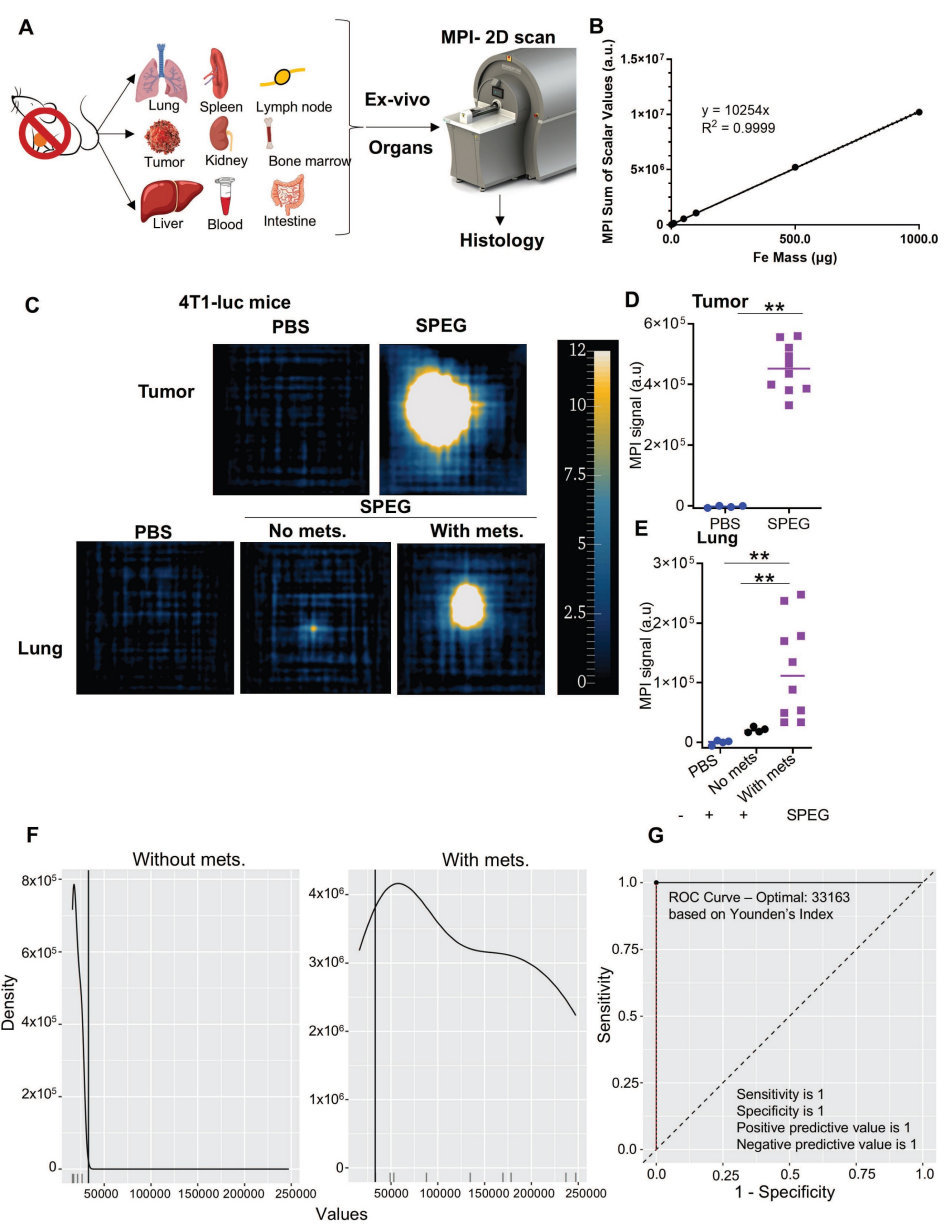
** Two-dimensional MPI analysis shows distinct accumulation of nanoparticles in tumors and lungs which can predict disease with high sensitivity and specificity.** (A) Organs of interest were dissected from mice after sacrifice and subjected to 2D MPI analysis followed by histology as illustrated. (B) A dose dependent linear increase in signal with total iron mass was noted with 2D analysis as well for SPEG particles (0.0005, 0.001, 0.005, 0.01, 0.05, 0.1, 0.5 and 1 mg Fe). (C) Tumors and lungs dissected from 4T1-luc tumor bearing and non-tumored mice sacrificed after live imaging were subjected to 2D scanning in MPI. The ex vivo tumors and lungs were scanned at a higher excitation field amplitude (10 mT) than *in vivo* (5 mT), in order to enhance the signal intensity. Tumors and lungs dissected from mice without any nanoparticle injection served as a control for 2D analysis. *Ex vivo* MPI scans of tissue from 4T1-luc mice 50, 11 and 77 are shown to represent the PBS-injected tumored mouse, SPEG-injected mouse with no metastases, and SPEG-injected tumored mouse with metastases, respectively. (D) A significantly high MPI signal was detected in tumors of SPEG injected mice. (E) Compared to PBS or SPEG injected mice lungs without metastasis (non-tumored mice), SPEG injected mice lungs with metastasis had significantly higher MPI signal in 2D *ex vivo* analysis. (F) Statistical analysis shows a distinct difference in the distribution of predictor (MPI) values by outcome for mice without and with metastasis. (G) ROC curve. The optimal cutoff value of 33163 (corresponding to an iron mass fo 3.2 µg) yields specificity of 1 (95%CI: [0.40, 1.00]), sensitivity of 1 (95%CI: [0.69, 1.00]), positive predictive value of 1 (95%CI: [0.69, 1.00]) and negative predictive value of 1 (95%CI: [0.40, 1.00]). All data points from individual mice are shown with median (Mann-Whitney **p < 0.01).

*Ex vivo* two-dimensional analysis enabled us to individually measure the MPI signal from each excised organ of interest, especially lungs, which had signal interference from the nearby liver and other metastatic nodules in the chest area. Each tissue was imaged with MPI using a sequence that measured the total-cumulative intensity within a 2D plane from each sample (Figure 2A). Two-dimensional analysis of SPEG also showed a linear relationship between MPI signal and particle mass, which was used as a calibration curve to quantitatively measure iron concentration from *ex vivo* organs (Figure 2B). Blood (0.1 mL) showed no signal, which indicated that the nanoparticle clearance was below detection limits after 72 h. Like 3D *in vivo* imaging data, 2D *ex vivo* data revealed higher signals in the liver, spleen, bone marrow, lymph nodes, kidney, and intestine in all models injected with SPEG (Figure S12, S13). Metastatic lungs and sternums of 4T1-luc mice (due to chest wall infiltrating tumors) had significantly higher MPI signal than non tumored mice (Figure S12). The spleens also had higher signal in 4T1-luc mice due to splenomegaly reported for this model 55. We also conducted a thorough histological evaluation for any organ toxicity in both mouse models after sacrifice. No indication of any toxicity was observed (Figure S12, S13).

We qualitatively observed *in vivo* that the max MPI signal intensities of micrometastases (lymph node, bone, eye region, etc) were similar to those in primary tumors (Figure 1C); however, quantitative analysis of the total MPI signal (sum of scalar values) from the 2D *ex vivo* organs showed ~4x higher total signal in primary tumors than in the micrometastases (Figure S14). Since the primary tumors are much larger, they can retain significantly more particles than the smaller metastatic nodules. MPI scans of the excised tumors and lungs from 4T1-bearing mice showed a high MPI signal, similar to 3D imaging *in vivo*, when compared to PBS controls or non-tumored SPEG injected mice. (Figure 2C-E and Figure S15). To determine whether the MPI signal alone could distinguish between SPEG mice with and without lung metastasis, we conducted a cutoff analysis aimed at maximizing the Youden index. A threshold of 33,163 (corresponding to a SPEG iron mass of 3.2 µg Fe) was identified from the full dataset. This cutoff was selected in 670 out of 1,000 bootstrap iterations, indicating moderate consistency across samples (Figure 2F, Figure S16, S17). At this threshold, the full dataset yielded a sensitivity, specificity, positive predictive value (PPV), and negative predictive value (NPV) of 1.00, with wide 95% confidence intervals reflecting the limited sample size (e.g., specificity 95% CI: [0.40, 1.00]; sensitivity 95% CI: [0.69, 1.00]) (Figure 2G and Table S2).

**Figure 3:**
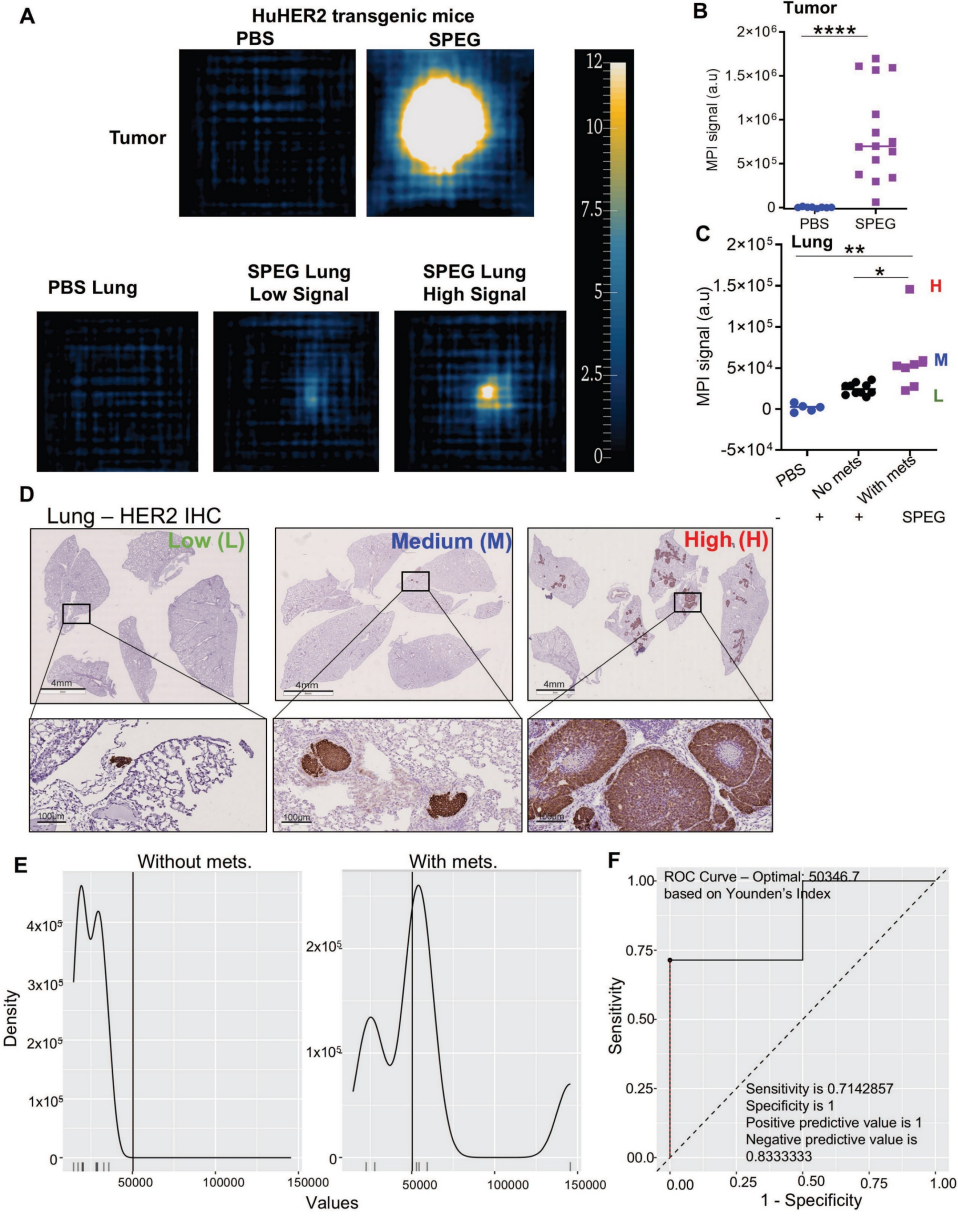
** Two dimensional MPI analysis distinguishes lungs with and without micrometastases in HuHER2 mice.** (A-C) In HuHER2 mice, SPEG tumors (mice n = 11, tumor n = 15) had a higher signal compared to PBS control tumors (mice n = 5, tumor n = 8). Lungs, when subjected to analysis, showed that SPEG-injected mice with metastatic nodules had a significantly higher MPI signal than those without metastasis or PBS-treated mice. A varying signal intensity was noted in the mice with metastasis group, indicated as high (H), medium (M) and low (L) signal. *Ex vivo* MPI scans of tissue from HuHER2 GEMMs 86, 962 and 192 are shown to represent the PBS-injected tumored mouse, SPEG-injected tumored mouse with low signal, and SPEG-injected tumored mouse with high signal, respectively. (D) HER2 IHC showed micrometastases of varying size and number that matched with the lung MPI signal intensity (Scale bar 4 mm and 100 µm), where HuHER2 GEMMs 955, 192, and 55 represent the lungs with low, medium, and high metastatic burden, respectively. (E) Statistical analysis showing the distinction of MPI signal distribution in mice without and with metastasis is given. (F) ROC curve. The optimal cutoff value of 50346.7 (corresponding to an iron mass fo 4.9 µg) yields specificity 1 (95%CI: [0.69, 1.00]), sensitivity of 0.71 (95%CI: [0.29, 0.96]), positive predictive value of 1 (95%CI: [0.48, 1.00]) and negative predictive value of 0.833 (95%CI: [0.52, 0.98]). All data points from individual mice are shown with median (Mann-Whitney *p < 0.05, **p < 0.01, ****p < 0.0001).

Next, we analyzed the huHER2 transgenic tumors, lungs and tissues. Additional 2D *ex vivo* data were collected from tumor bearing mice (n = 11 total). Signal obtained from 2D *ex vivo* MPI of tumors showed a high MPI signal when compared against PBS injected controls (Figure 3A-B, Figure S18). Unlike the 4T1-luc model, the 2D* ex vivo* MPI signal measured from the lungs of the huHER2 mice was comparatively lower (about 100-fold). Lung tissues were stained with a human anti-HER2 antibody via immunohistochemistry (IHC) to unambiguously confirm the presence of mammary-derived HER2^+^ cell clusters (micrometastatic tumors) in lungs (Figure S19). We observed a significant difference in MPI signal between the *ex vivo* lungs of mice with and without metastasis (Figure 3C). Some lungs with metastatic disease had an MPI signal comparable to non-metastatic lungs. Further investigation revealed that those lungs, which we denote as having low signal (L), had < 3 micrometastatic nodules when compared to lungs with higher signal, which are categorized as either medium (M) or high (H) (Figure 3D, Figure S19). Statistical analysis of this cohort using the MPI signal as a diagnostic marker identified a threshold of 50,347 (corresponding to a SPEG iron mass of 4.9 µg Fe), based on maximizing the Youden index. At this threshold, the full dataset yielded a specificity of 1.00 (95% CI: [0.69, 1.00]), sensitivity of 0.71 (95% CI: [0.29, 0.96]), positive predictive value (PPV) of 1.00 (95% CI: [0.48, 1.00]), and negative predictive value (NPV) of 0.83 (95% CI: [0.52, 0.98]). The Youden index for this threshold was 0.71 (Figure 3E-F, Figure S20-21, and Table S3). We also conducted a combined analysis incorporating results from both models, which identified a threshold of 33,163 (corresponding to 3.2 µg Fe), selected in 466 out of 1,000 bootstrap iterations. This threshold yielded a specificity of 0.93 (95% CI: [0.66, 1.00]) and sensitivity of 0.88 (95% CI: [0.64, 0.99]), with a PPV of 0.94 (95% CI: [0.70, 1.00]) and NPV of 0.87 (95% CI: [0.60, 0.98]) for distinguishing between mice with and without lung metastasis (Figure S22-24, Table S4). While these findings suggest that the MPI signal may have strong discriminatory potential, they remain preliminary and should be interpreted with caution due to wide confidence intervals around sensitivity and specificity estimates. A larger cohort would provide greater precision and statistical power.

### Nanoparticles are retained in the tumor microenvironment

**Figure 4:**
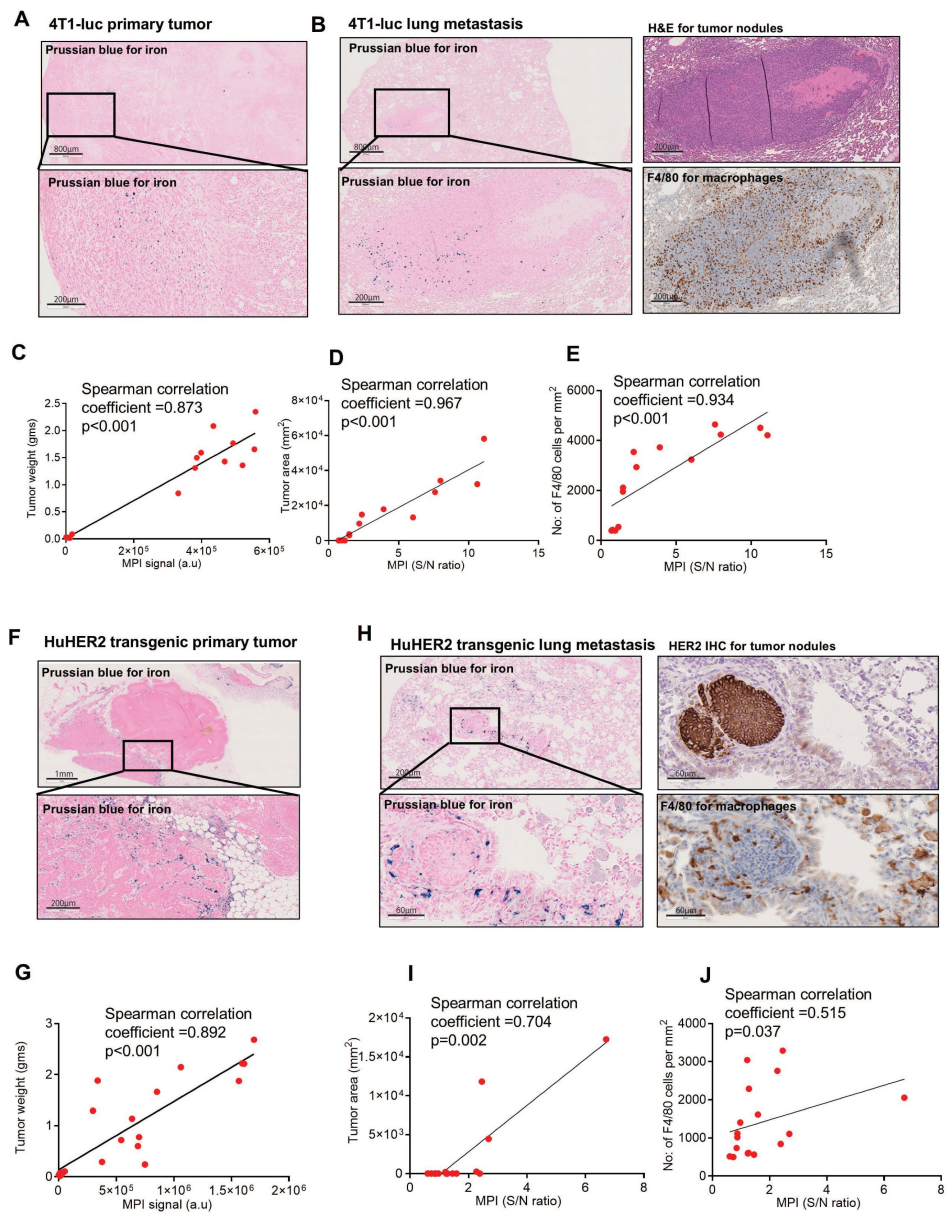
** MPI signal intensity correlated positively with primary tumor, metastatic area and number of macrophages in TME.** (A) Prussian blue staining of 4T1-luc (Mouse 49) tumor shows the presence of nanoparticles in the 4T1-luc tumor microenvironment. (Scale bar 800 µm and 200 µm). (B) Prussian blue analysis of 4T1-luc (Mouse 77) lung shows nanoparticle presence in the tumor nodules (Scale bar 800 µm and 200 µm). Tumor nodules were identified with H&E and macrophages by F4/80 staining (Scale bar 200 µm). (C) A correlation analysis between tumor weight and 2D MPI signal shows a strong positive correlation. (D) A correlation analysis of the MPI signal to metastatic tumor nodule area within lungs and (E) number of macrophages in lungs identified by F4/80 staining showed a highly positive correlation. (F) Similarly, by Prussian blue staining, HuHER2 transgenic tumors (Mouse 962) also showed nanoparticles in the tumor microenvironment (Scale bar 1 mm and 200 µm). (G) Here too, there is a strong positive correlation between tumor weight and MPI signal. (H) HuHER2 lung metastatic nodules from Mouse 192 identified by HER2 IHC showed Prussian blue positivity, indicating nanoparticle presence (Scale bar 200 µm and 60 µm). Nanoparticle presence surrounding the growing tumor nodules coincided with the macrophages identified by F4/80 staining (Scale bar 60 µm). (I & J) HuHER2 mice lung MPI signal also correlated with metastatic nodule area in the lung and also with the number of macrophages in the lung identified as F4/80 positive cells.

To exploit the utility of nanoparticle-mediated metastasis detection using MPI, we needed to understand the localization of nanoparticles and their correlation with the tumor area in metastatic sites. The presence of nanoparticles in the TME of both primary tumors and lung metastases (identified from H&E slides) in 4T1-luc tumor-bearing mice was confirmed by Prussian blue staining (Figure 4A-B). Our subsequent analysis revealed a highly positive correlation (Spearman correlation coefficient ρ = 0.873, p < 0.001) between *ex vivo* MPI signal and primary tumor mass (Figure 4C). Contralateral non-tumored mammary glands from SPEG injected mice served as controls for the background signal. The analysis also revealed a strong correlation (ρ = 0.967, p < 0.001) between the measured *ex vivo* MPI signal-to-noise (S/N) ratio and tumor area in lungs (Figure 4D). We also observed F4/80^+^ macrophages in Prussian blue+ nanoparticle areas in lung tissue. Further analysis revealed a strong positive correlation (ρ = 0.934, p < 0.001) with the total number of F4/80 macrophages and MPI signal in the *ex vivo* lungs (Figure 4E). Similarly, in huHER2 GEMMs, Prussian blue^+^ nanoparticles were seen in the primary tumors (Figure 4F) and a strong positive correlation (ρ = 0.892, p < 0.001) between primary tumor weight and *ex vivo* MPI signal was observed (Figure 4G). In the lungs, nanoparticles were seen surrounding micrometastases identified by HER2 staining and were associated with macrophages (Figure 4H). Here too, analysis revealed a positive correlation between *ex vivo* MPI signal and metastatic nodule area (Figure 4I) (ρ = 0.704, p = 0.002) and number of macrophages (Figure 4J) in lungs (ρ = 0.515, p = 0.037).

Overall, this shows that tumor-associated macrophages (TAMs) were responsible, in part, for nanoparticle retention in the TME and tumor periphery. This was more apparent in huHER2 tumors (Figure S25) which displayed more extensive stromal regions than 4T1-luc tumors. Additional IHC staining revealed an association between nanoparticles and a variety of stromal and immune cell types in huHER2 tumor sections than the HER2^+^ tumor cells (Figure S26). From the results of those analyses, we can conclude that tumor-associated macrophages (F480), fibroblasts (α-SMA), dendritic cells (CD11c), endothelial cells (CD31), and collagen (Masson's trichrome) all contributed to nanoparticle retention, whereas no HER2^+^ correlations with nanoparticles were observed, similar to previously reported observations 18,19, 56.

When we compared the *ex vivo* lung MPI signal of both models, there was a difference in the overall MPI signal in HuHER2 lungs. This was mainly due to the difference in the metastatic burden in these two models. Higher tumor burden in 4T1-luc lungs had an increased number of macrophages, thereby contributing to higher uptake of nanoparticles, resulting in higher MPI signal (Figure S27). Nanoparticle retention in the tumor region is widely debated, with recent observation of active receptor mediated uptake and retention along with enhanced permeability and retention due to vascular leakage 36. Here, we observed nanoparticle retention in tumor-associated stromal and immune cells even within micrometastatic regions. Further detailed studies are necessary to evaluate the mechanism of retention.

### Translation to human-scale MPI for nanoparticle detection

Immune cell phenotype and function are known to vary by location within the TME, as part of the tumor core and towards the infiltrating edge 57. In both the 4T1-luc model and huHER2 GEMM, we detected high accumulation of nanoparticles predominantly at the tumor periphery by MPI (Figure 1C-D) and Prussian blue staining (Figure S25). TAMs in the periphery tend towards an anti-inflammatory phenotype and support tumor expansion and intravasation 58, 59. Intratumoral localization of immune cells can serve as a clinical biomarker and has been linked to treatment responses 60, but it is difficult to determine non-invasively.

**Figure 5:**
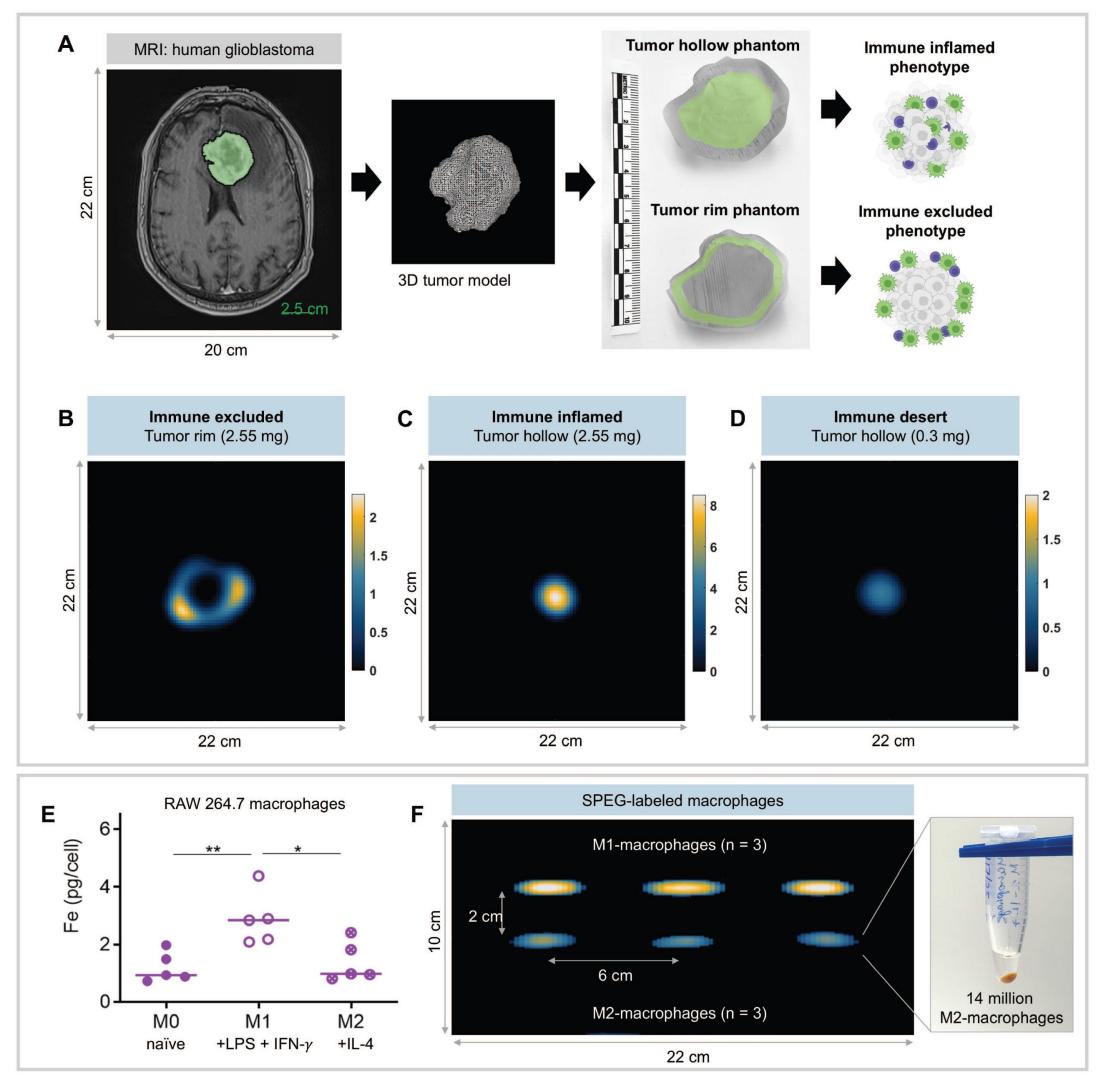
** Human-sized tumor phantoms demonstrate the potential for imaging TAMs with a human-scale MPI scanner.** (A) A contour of a human glioblastoma was segmented from an MRI (20 x 25 x 5.6 cm) 30, 31. After generating a 3D model, two tumor phantoms were created with a fillable region consisting of a hollow core or a 3-mm wide rim, to represent immune-inflamed and immune-excluded phenotypes, respectively. (B) MPI of the tumor rim phantom filled with 2.55 mg SPEG is visually distinct from the tumor hollow phantom (C) due to the peripheral distribution of SPEG. (D) Human-scale MPI shows sensitive detection of 0.3 mg SPEG in the tumor hollow phantom. The MPI signal in this phantom (0.3 mg SPEG) was 8.5× lower than for 2.55 mg SPEG, indicating linear scaling of MPI values with the amount of SPEG. 3D MPI dimensions of 22 x 22 x 20 cm are displayed as a 5-mm thick slice. (E) Murine macrophages in different inflammatory states show differences in SPEG uptake after *in vitro* incubation, with on average 0.883 pgFe/cell for naïve (M0) macrophages, 2.184 pgFe/cell for pro-inflammatory (M1) macrophages, and 0.956 pgFe/cell for anti-inflammatory (M2) macrophages by ferene assay (five independent experiments, all data points with median is shown - *p < 0.05, **p < 0.01). (F) MPI detection of SPEG labeled M1 and M2-macrophage cell pellets separated by 2 cm, containing 14 million cells (n = 3) using a human-scale MPI device. The corresponding iron concentration in cell pellets was 30.6 µg for M1 cells (2.184 pgFe/cell) and 13.4 µg for M2 cells (0.956 pgFe/cell).

To explore the potential for imaging TAMs in human tumors with a human-scale MPI device 61, 62, volumetric glioblastoma tumor phantoms (Movie S7) were derived from a patient MRI 30, 31. Here, we focused on a glioblastoma phantom because the clinical MPI system currently has a field of view optimized for brain imaging and because macrophages are also abundant in glioblastomas and brain metastases, where their presence correlates with poor prognosis 63, 64. To replicate the nanoparticle-rich TME at the tumor periphery (an immune-excluded tumor), one phantom includes a 3-mm rim at the tumor edge and the other has a fillable/hollow core representing an immune-inflamed tumor with TAMs distributed throughout (Figure 5A). The tumor phantom in Figure 5 does not capture the cellular complexity of the tumor microenvironment and does not distinguish nanoparticle uptake by immune cell subsets, tumor cells, or stromal elements. This phantom was intended as a proof-of-concept to demonstrate the feasibility of MPI detection on a human scale.

The tumor phantoms were filled with 2.55 mg SPEG to represent ~ 0.5% of a hypothetical, clinically-relevant injected dose of 510 mgFe 65-68. The value of 0.5% was chosen based on the accumulated SPEG dose in mouse lungs with confirmed metastases (Figure S15, S18). MPI scans of the tumor rim phantom produced a visually distinct annular distribution, similar to our *in vivo* tumor mouse imaging results (Figure 5B, Figure S28). This is visually distinct from the tumor hollow phantom that shows uniform signal distribution (Figure 5C). To demonstrate an immune desert tumor and assess the sensitivity of the human-scale MPI scanner, the tumor hollow phantom was reimaged with substantially fewer particles, 300 µg Fe or 0.059% of a 510 mg Fe clinical dose, which resulted in an 8.5× reduction in MPI signal (Figure 5D). MPI can detect SPEG at spatially varying regions in tumor phantoms, supporting the future use of MPI to non-invasively quantify and assess the distribution of nanoparticle engulfed cells in the TME non-invasively at the human scale.

Mouse and human breast tumors contain a spectrum of M0-, M1-, and M2-like macrophages, with predominant anti-inflammatory (M2) phenotype in aggressive subtypes 69. To test whether nanoparticle uptake depends on the specific phenotype of scavenging macrophages, we tested murine macrophages isolated from the ascites of tumor bearing mice. When induced to their proinflammatory (M1) phenotype with LPS and IFN-γ, RAW267.4 macrophages engulfed significantly more nanoparticles (2.8 pg/cell) than either naïve M0 macrophages (0.93pg/cell) or anti-inflammatory M2 macrophages (0.96pg/cell) (Figure 5E). This *in vitro* assay provides an estimate of relative *in vivo* labeling efficiency and confirms the expectation for TAMs of each phenotype to contribute to the MPI signal. After *in vitro* labeling with SPEG, we demonstrated detection of individual M1- and M2- induced macrophage pellets containing 14 million cells (n = 3) using the human-scale MPI device. This MPI scanner resolved M1- and M2-like macrophage pellets separated by 2 cm (Figure 5F). As few as 13.4 µg Fe in 14 million cell pellets (average uptake of iron per M2 macrophage = 0.96 pg) was detected, representing 0.0026% of the typical dose for i.v. Ferumoxytol in humans (510 mg Fe). These images show the potential to image macrophages in tumors and metastatic sites at the human scale with sufficient cellular sensitivity and resolution. Distinguishing whether cell type or activation state governs the total mass of nanoparticles ingested by an individual cell remains to be determined. Although our *in vitro* studies confirmed that M0, M1, and M2 macrophages can all load SPEG nanoparticles, *in vivo* macrophage phenotypes exist along a continuum and are compartmentalized within distinct regions of the TME that have varying access to circulating nanoparticles 69-72. As such, we did not attempt to distinguish phenotype-specific contributions to the MPI signal in Figure 4.

Radiotracer studies using the 4T1 model have not shown promising results in identifying metastatic lesions accurately in regions like lymph nodes and adipose tissue in the leg as shown in this study 73-75. Prior studies in the 4T1 model underscore the limitations of established approaches. Iron-based MRI generates negative contrast that is difficult to interpret in organs like air-filled lungs and ^18^F-FDG PET has shown limited ability to sensitively and specifically detect micrometastases 76, 77. These comparisons suggest MPI may offer advantages by providing positive contrast and specific detection of iron-labeled inflammatory and tumor-associated cells. Our findings are consistent with and extend prior preclinical work demonstrating that MPI can sensitively detect metastatic disease, including breast cancer lymph node metastases and prostate cancer bone metastases 15, 78. Together, these studies highlight the versatility of MPI for detecting diverse metastatic sites across cancer types.

Before widespread adoption, a notable technical challenge for MPI is the shine-through problem that occurs when imaging signals of high dynamic range are in close proximity 76, 79, 80. As shown in mouse models, infused SPEG nanoparticles accumulate significantly in naturally macrophage-rich organs, particularly the liver and spleen 81. Due to intense interference with the MPI signal in these organs, there is a limited capability to detect tumors and metastasis containing lower SPEG concentration from nearby tissues such as the lungs. To investigate this dynamic range problem, preliminary computational simulations for the Momentum MPI system were conducted for two nanoparticle sources for 3D MPI. As the dynamic range increased, the required distance between nanoparticle sources to enable their resolution also increased (Figure S29A). Experimentally, we demonstrate that two-point sources with a 10x concentration difference can be resolved < 5mm in distance using a shine-through phantom and 2D MPI (Figure S29B). The SPEG concentration used for phantom imaging represents the amount of SPEG in the mouse liver and metastatic lung.

To mitigate the dynamic range challenge from strong liver and spleen signals, we employed two strategies in this study. First, PEGylation of Synomag® nanoparticles prolonged circulation time and reduced immediate sequestration by the reticuloendothelial system (RES), improving contrast between clearance organs and tumors. Second, we implemented an iterative physics-based image reconstruction approach that improved resolution in high dynamic range. Future studies are necessary to further evaluate the limitations and mitigation strategies of MPI detection and quantification in these high dynamic range settings using relevant models. The dynamic range issue in MPI will eventually be overcome by combinations of new tracer technology, hardware advances, and improved image reconstruction algorithms. Furthermore, future work may include sensitivity analysis for detecting early lesions while optimizing mouse models, tracer design, dose, and timing for injection. Extensive efforts are underway to engineer next-generation MPI nanoparticles with optimized magnetic properties and multifunctionality 82, 83, which may further enhance the sensitivity and resolution for MPI.

This work provides evidence that MPI can be used to quantify inflammatory components of tumors and metastases that enabling their detection in live subjects without using a radioactive tracer. MRI detection of TAMs has been successful in several pilot studies in humans using ferumoxytol, showing nanoparticle accumulation at the tumor periphery in aggressive glioblastomas 65. These studies show the ability to support the diagnosis of tumor subtypes based on the inflammatory component and to predict immunotherapy response for brain metastasis 64-66. However, iron-based MRI artifacts (signal voids) are challenging to interpret due to non-linear quantitation and are confounded by the presence of intratumoral hemorrhage 65. Prior ferumoxytol-MRI studies in glioblastoma and breast cancer brain metastases demonstrate that macrophages can be labeled *in vivo* with iron tracers, but quantification is challenging due to negative contrast 65-68. Our results highlight MPI's potential to overcome these limitations, providing a positive contrast with quantitative capability for future human applications. As a diagnostic tool, MPI could be used to distinguish pseudoprogression from radiation necrosis 84, which is challenging with existing imaging techniques. Direct detection of iron-labeled TAMs could be used to guide treatment strategy, such as to mark specific tumor areas for accurate biopsy targeting 67 and predict responses to immunotherapies 68, 85. MPI has the potential to monitor macrophage-targeted immunotherapies 67, 86-88 where increased accumulation of iron could be quantified in regions of suspected treatment effects. Furthermore, a similar MPI methodology could be applied to other inflammatory conditions to detect and quantify the immune infiltrates 67, 89.

## Conclusion

This study demonstrates the utility of magnetic particle imaging for the detection of tumors and metastases. This was achieved through iron oxide nanoparticle uptake and retention by stromal and immune cells in the tumor microenvironment, especially tumor associated macrophages. We are hopeful that MPI can serve as a useful modality to diagnose a variety of inflammatory conditions, in addition to cancer.

## Supplementary Material

Supplementary figures and tables, movie legends.

Supplementary movies.

## Abbreviations

µCT: micro computed tomography
^18^F-FDG)/PET: ^18^F-fluorodeoxyglucose/positron emission tomography
BLI: bioluminescent imaging
CD: cluster of differentiation
CI: confidence interval
CT: computed tomography
Fe: iron
GEMM: genetically engineered mouse model
H&E: hematoxylin & eosin
HER2: human epidermal growth factor receptor 2
HuHER2 model: human epidermal growth factor receptor 2 overexpressing breast cancer model
IHC: immunohistochemistry
MIP: maximum intensity projections
MPI: magnetic particle imaging
MRI: magnetic resonance imaging
NPV: negative predictive value
PBS: phosphate buffered saline
PEG: polyethylene glycol
PET: positron emission tomography
PPV: positive predictive value
SPEG: polyethylene glycol coated synomag®
RES: reticuloendothelial system
TAMs: tumor associated macrophages
TME: tumor microenvironment
TNBC: triple negative breast cancer
α-SMA: alpha- smooth muscle actin
