# Supplementary material for: Nanoparticle retention enables non-invasive detection of metastases by magnetic particle imaging in murine breast cancer models
Source: Theranostics. 2026 Jan 1;16(5):2136–55. doi: 10.7150/thno.122259 (PMC12712802; doi:10.7150/thno.122259)
Supplement: Supplementary file 1 — Supplementary figures and tables, movie legends. [file thnov16p2136s1.pdf]

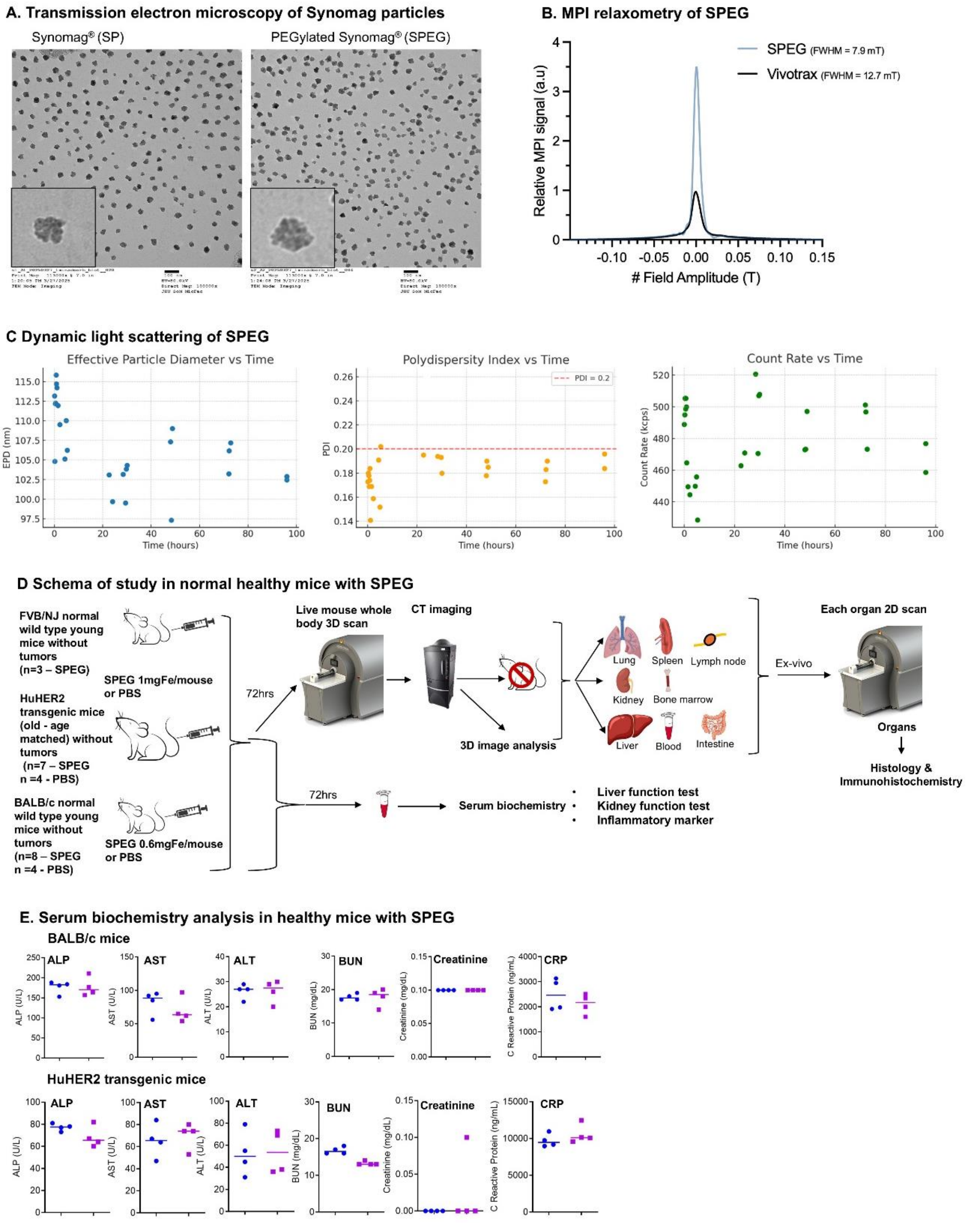

Figure S1: In vitro and in vivo characterization of SPEG. (A) Transmission electron microscopy (TEM) images of base Synomag® nanoparticles compared with Pegylated Synomag®. Both show core nanoflower

structure. (B) MPI relaxometry shows SPEG particle produces 3.5x maximum signal compared to Vivotrax (Magnetic Insight Inc), and SPEG MPI resolution is improved by 1.6x compared to Vivotrax. (C) Dynamic light scattering (DLS) stability study of SPEG nanoparticles, measuring effective particle diameter, polydispersity index (PDI), and count rate over 92 hours. Plots show stable size (hydrodynamic diameter ~102-110 nm), PDI < 0.20, and consistent scattering intensity, suggesting minimal sedimentation. (D) Schema of experiment with non-tumored mice. (E) Serum biochemistry analysis of SPEG injected mice compared to PBS injected controls shows no apparent difference in any of the liver function parameters – alkaline phosphatase (ALP), aspartate transaminase (AST), alanine transaminase (ALT) or kidney function parameters – blood urea nitrogen (BUN) or creatinine. We also tested the inflammatory marker C-reactive protein (CRP) which also remained unchanged.

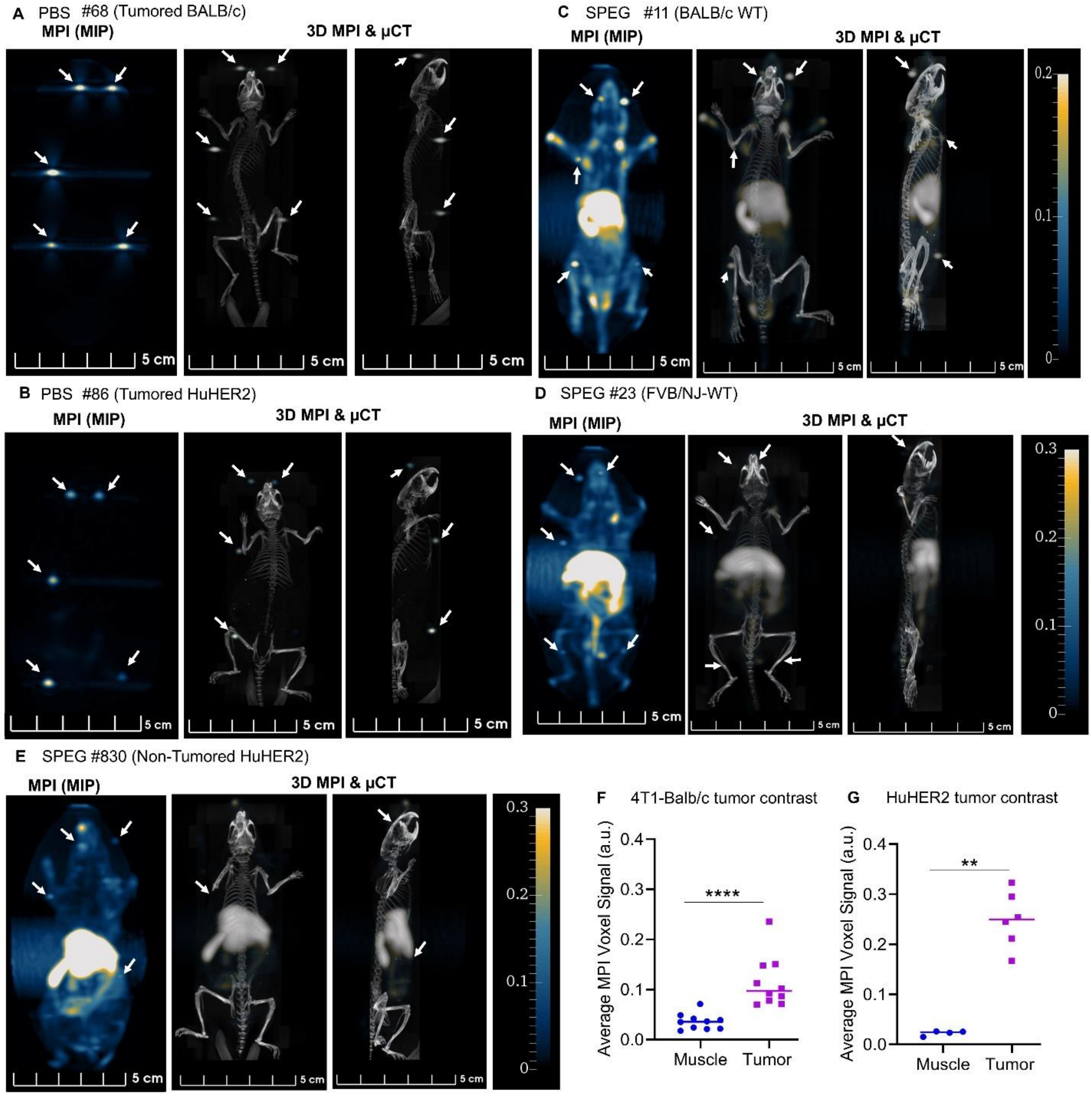

Figure S2: Representative MPI 2D projection and 3D scan with co-registered microCT of (A) Tumored BALB/c mouse injected with PBS (B)Tumored HuHER2 transgenic mouse injected with PBS (C) normal BALB/c mouse without tumor injected with SPEG (D) normal FVB/NJ mouse without tumor injected with SPEG and (E) age matched non-tumored HuHER2 transgenic mouse injected with SPEG. All mice were subjected to whole body 3D scanning in MPI after 72 h of injection. Visible fiducials are marked with white arrows.(F-G) Quantitation of ~30 mm^3 spherical volume from 3D data of the left leg muscle compared to a similar sized segmentation of tumors in the mice shows significant MPI signal in the tumor area showing a significant difference. All data points from individual tumors are shown with median (Mann-Whitney **p <0.01,****p <0.0001).

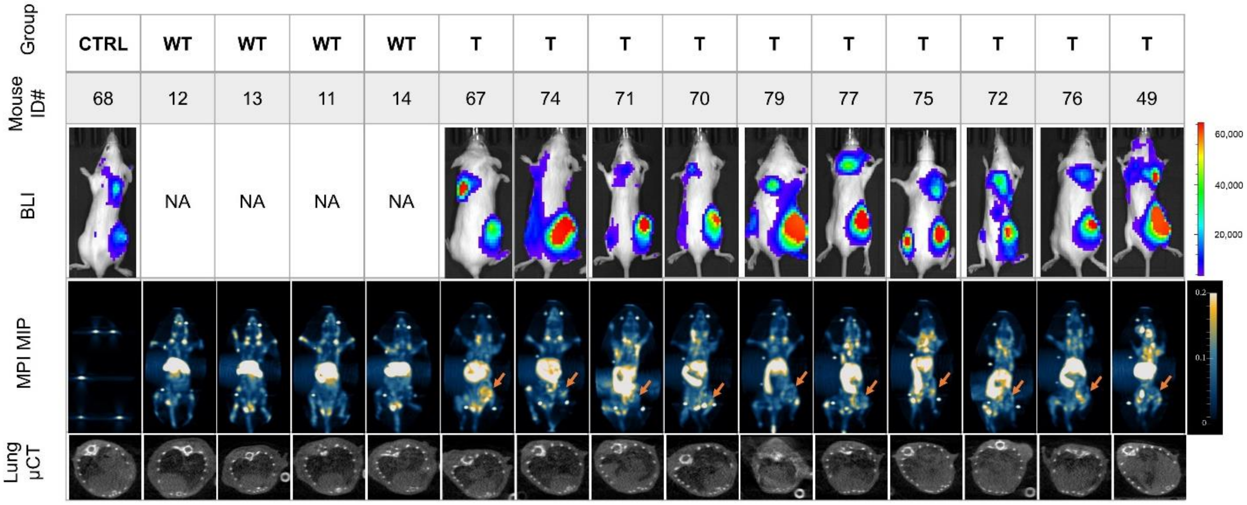

Figure S3: Images acquired from each BALB/c mouse with and without 4T1-luc tumor using bioluminescence (BLI), micro-computed tomography (µCT) or MPI (Magnetic Particle Imaging) as described in the Methods. Primary tumors are indicated using an orange arrow. (CTRL – 4T1-luc tumored mouse that received PBS injection, WT – SPEG injected normal non-tumored BALB/c mice, T – SPEG injected tumored mice, NA - not applicable).

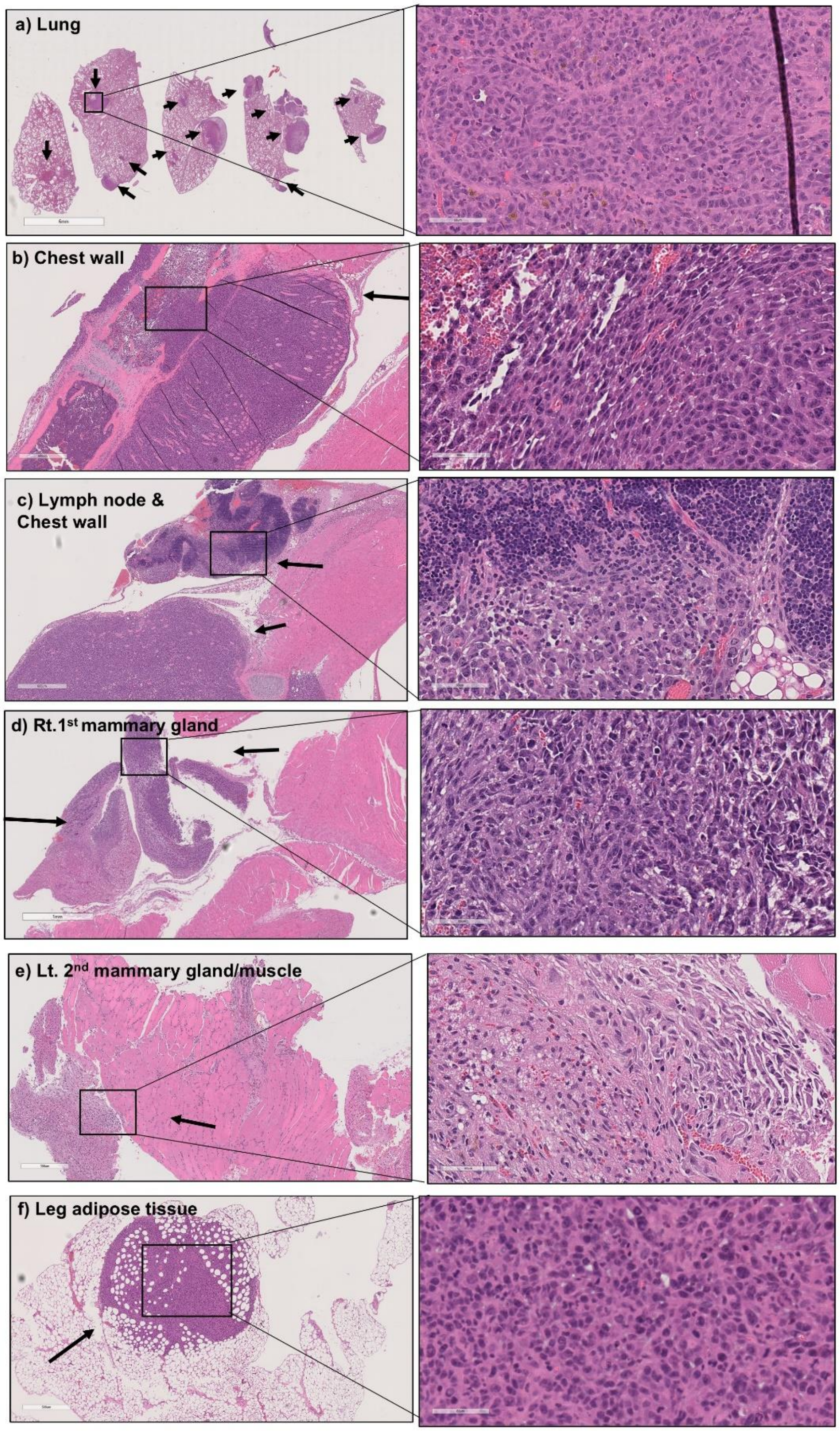

Figure S4: Histology analysis by H&E staining confirms the presence of metastasis in organs/areas identified through MPI in 4T1 Mouse 77 shown in Figure 1C.

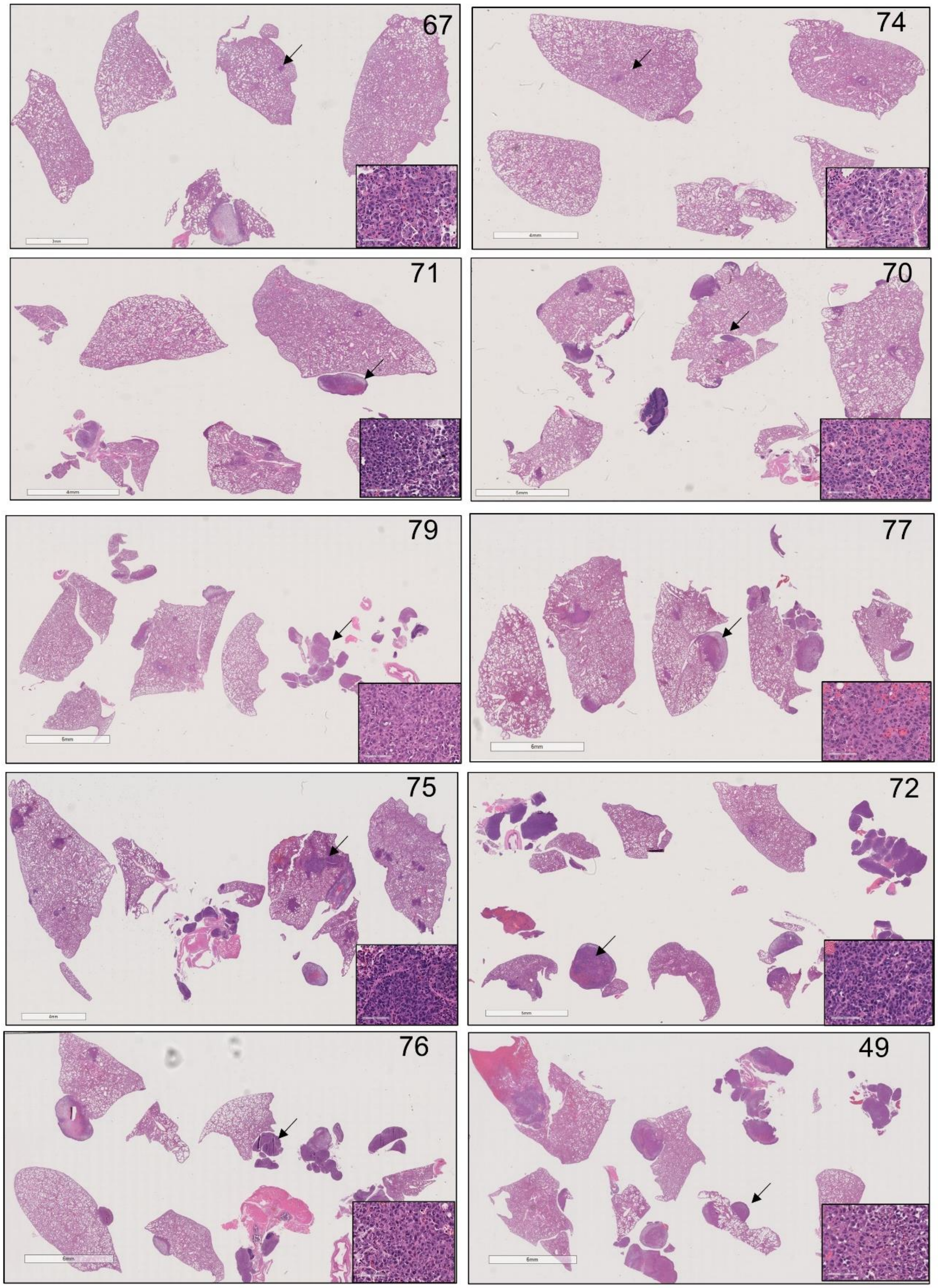

Figure S5: H&E stained lungs of individual 4T1-luc tumor bearing mice injected with SPEG showing metastatic nodules in the lungs. Inset shows enlarged image of a nodule from the lungs (black arrow).

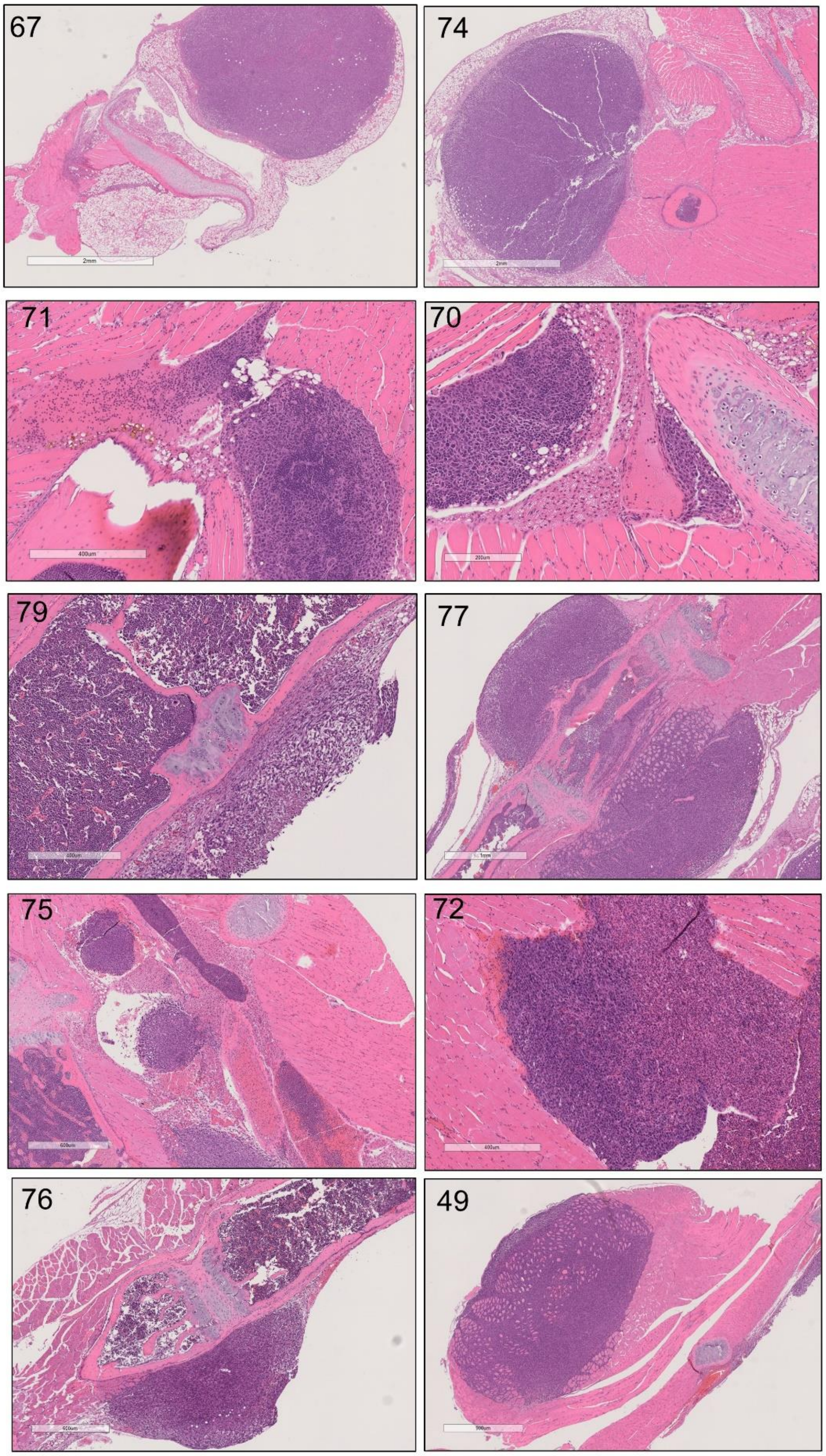

Figure S6: H&E stained chest wall of individual 4T1-luc tumor bearing mice injected with SPEG showing metastatic nodules infiltrating the muscles in the sternum/ribs.

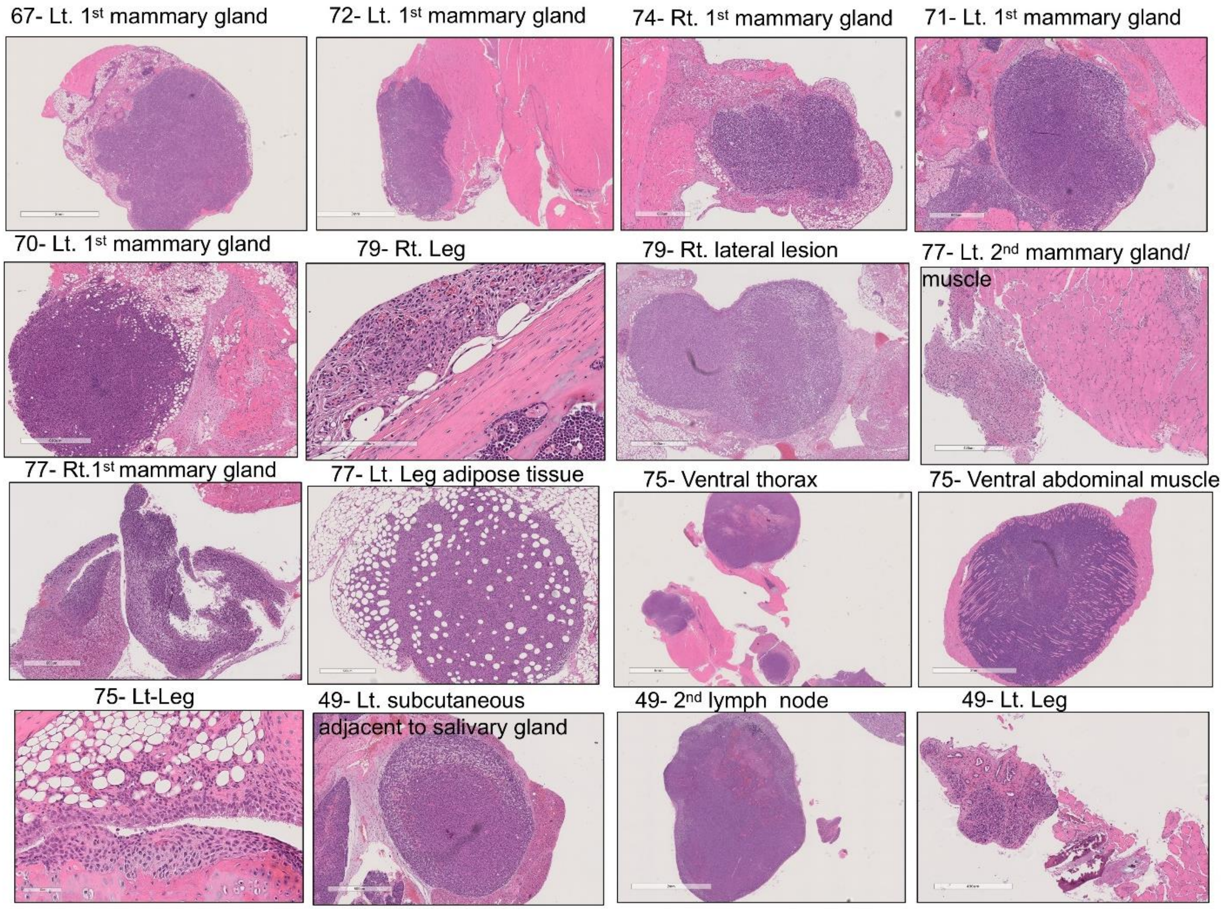

Figure S7: H&E stained tissues with MPI signal from individual 4T1-luc tumor bearing mice injected with SPEG confirm the presence of metastatic nodules.

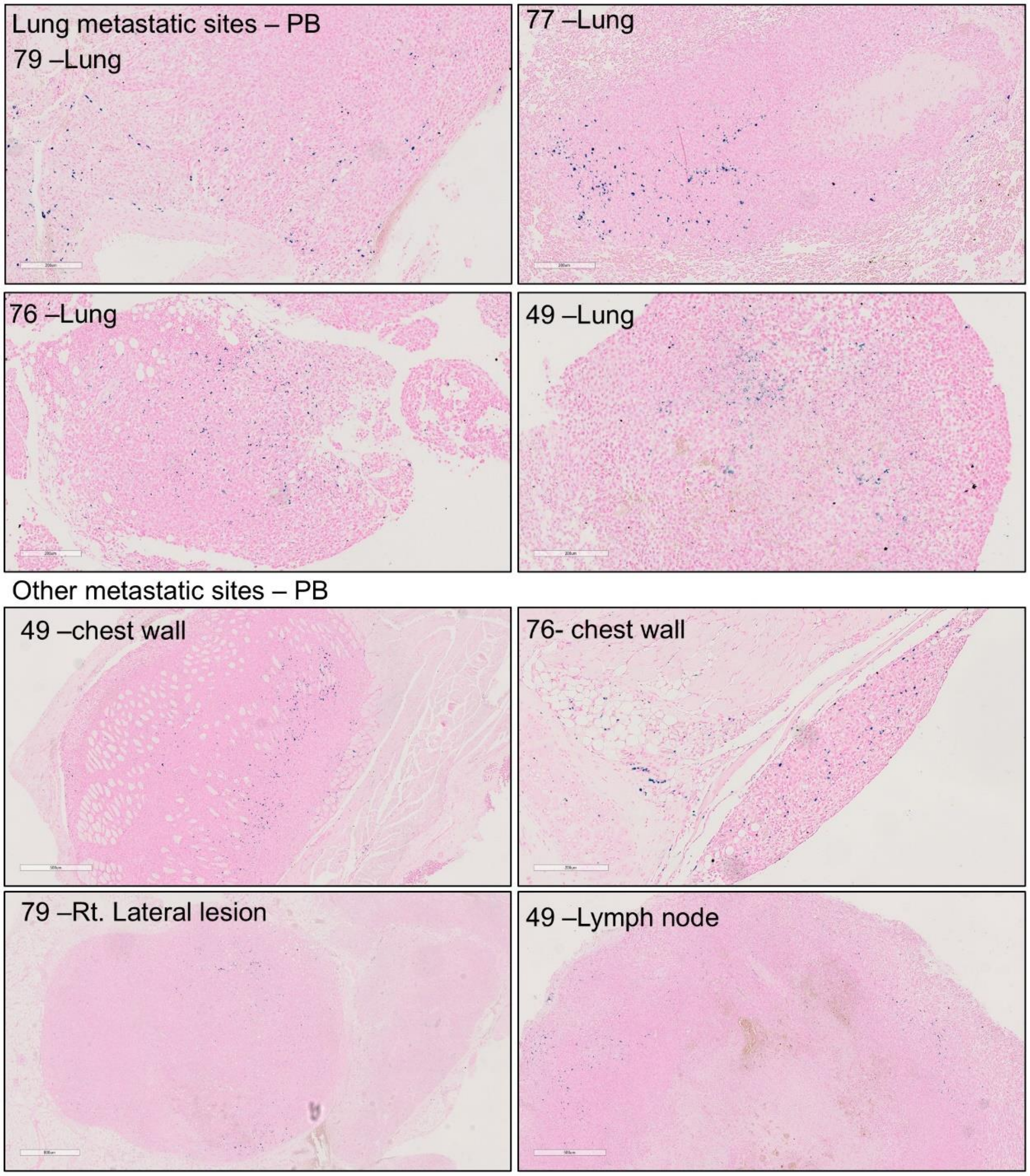

Figure S8: Representative Prussian blue stained slides showing nanoparticle accumulation in metastatic nodules of the lung and other sites from 4T1-luc tumor bearing mice injected with SPEG.

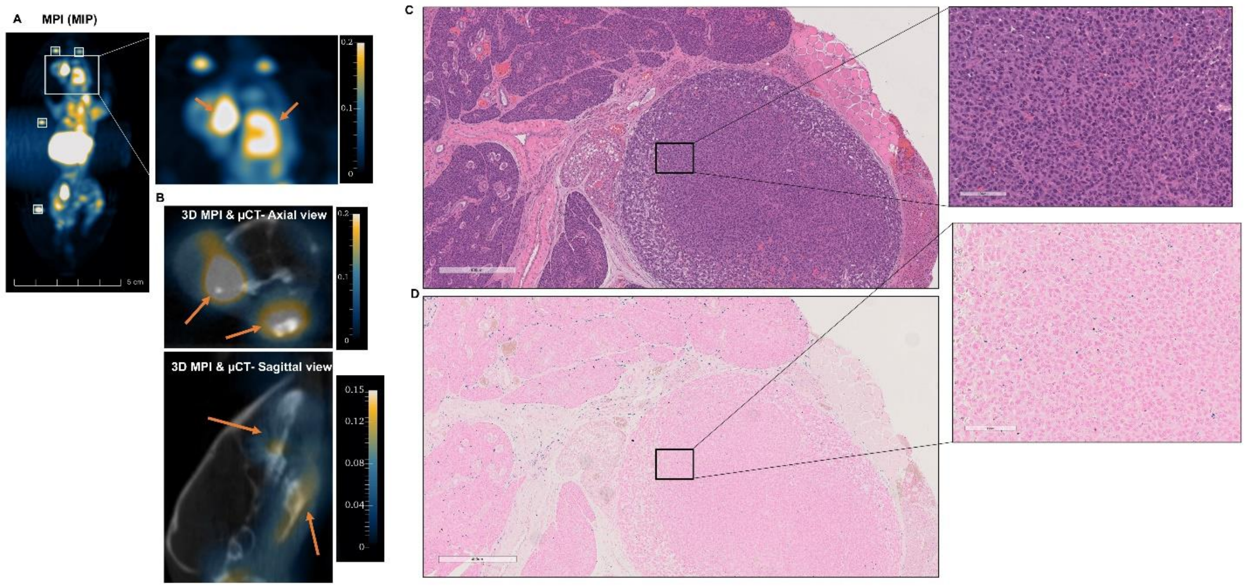

Figure S9: A) One mouse (#49), presented a positive MPI signal in its head area (orange arrows). B) Co-registration with μCT confirmed that the signals are arising from the area next to the left eye and from the opposite side of the brain. C) Histology analysis by H&E confirmed the presence of subcutaneous metastatic nodules adjacent to the salivary gland. D) Prussian blue analysis revealed nanoparticle presence in the metastatic nodule and surrounding stromal region in the salivary gland tissue as shown here.

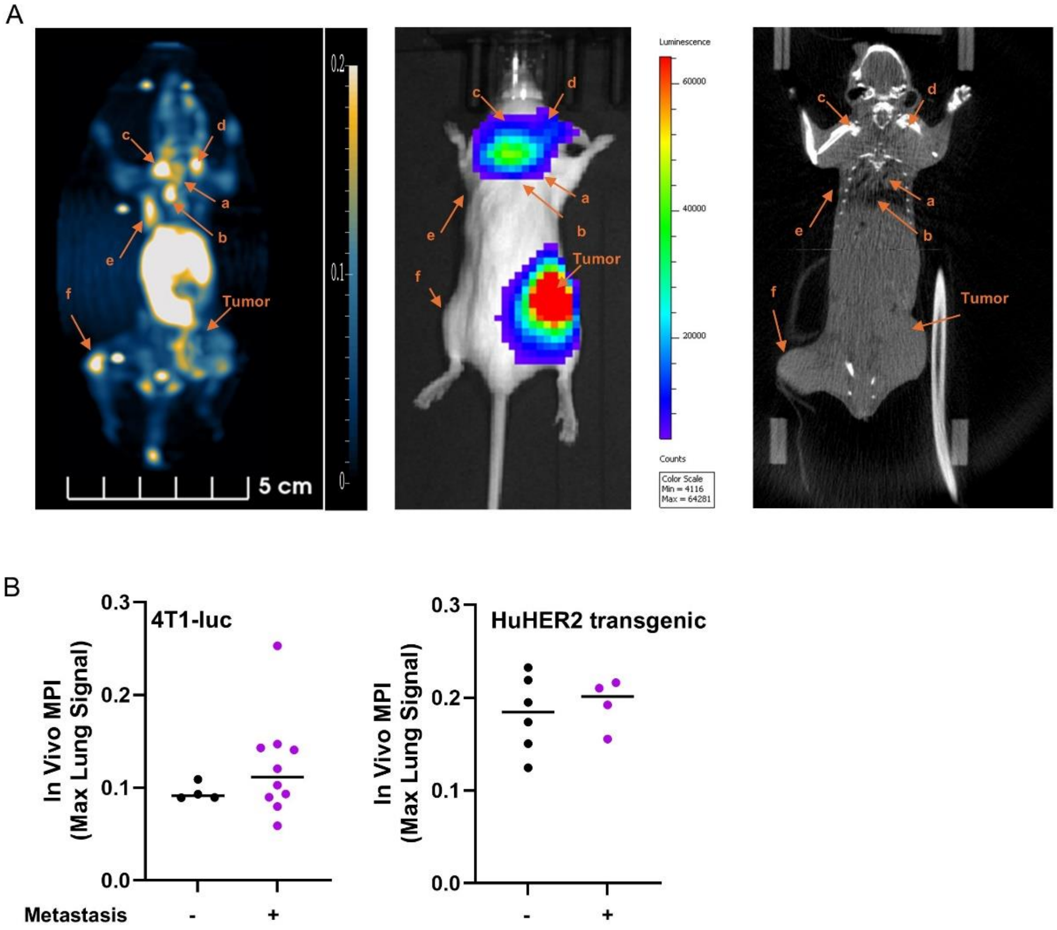

Figure S10: (A) Representative image of Mouse 77 with multiple micro metastases away from the lungs identified from MPI and confirmed with histology that did not show distinctly in BLI or µCT. (B) Quantitative analysis of the in vivo 3D lung after segmentation was obscured by the high signal interference from the liver.

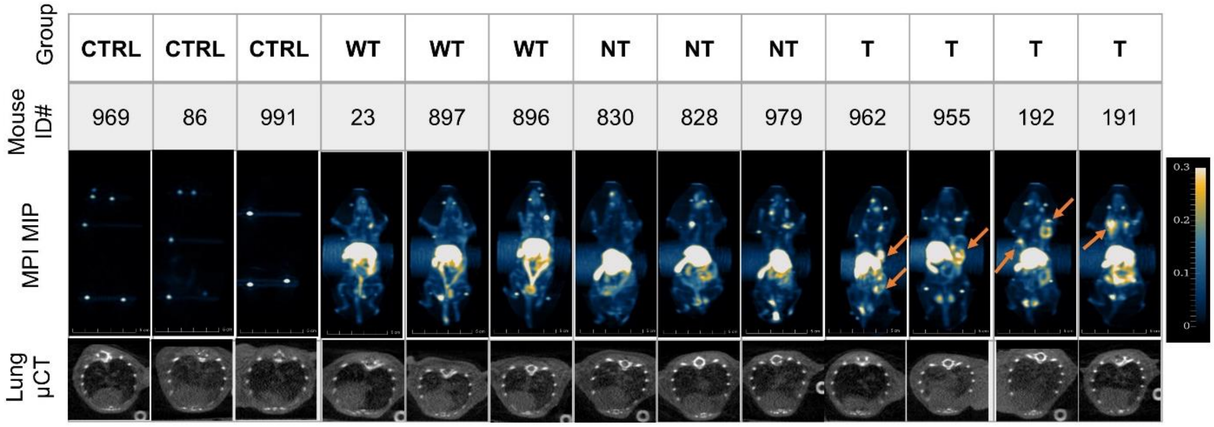

Figure S11: Shown are 2D projections and slices of 3D images acquired from each individual HuHER2 transgenic mouse with and without tumor or FVB/NJ normal mice using MPI (Magnetic Particle Imaging) and micro-computed tomography (μCT) as described in the Methods. Tumors are indicated by orange arrows. (CTRL – PBS injected tumored HuHER2 transgenic mice, WT – SPEG injected normal non-tumored FVB/NJ mice, NT- SPEG injected age-matched non-tumored HuHER2 transgenic mice, T – SPEG injected tumored HuHER2 transgenic mice).

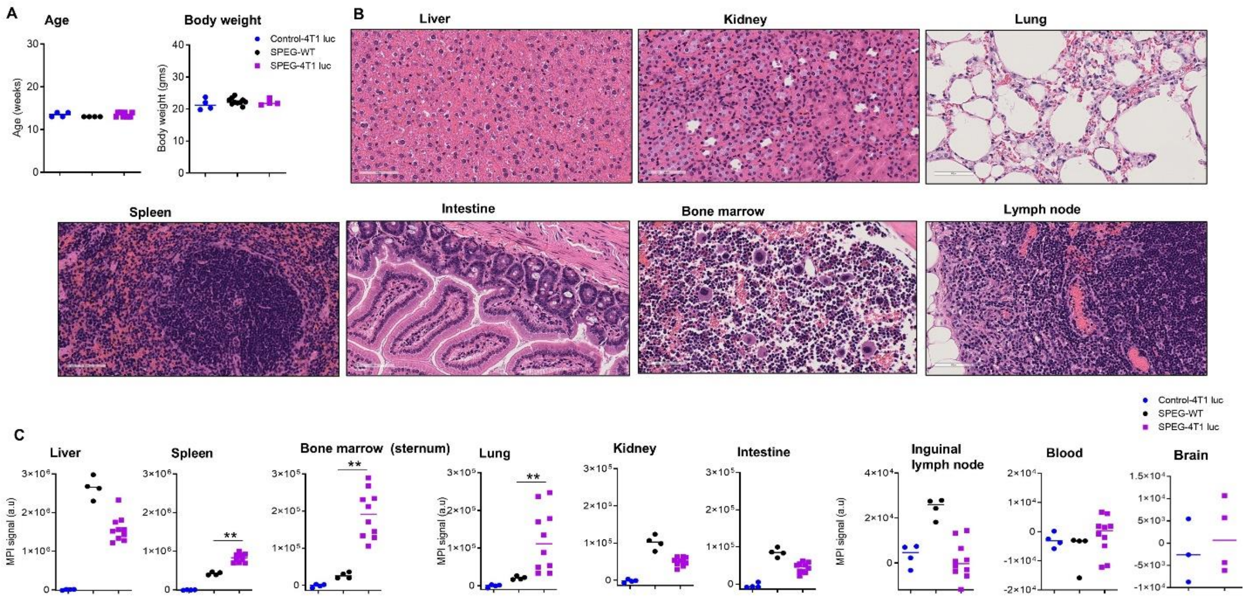

Figure S12: (A) Age and bodyweight of BALB/c mice (with and without 4T1-luc tumor) used in this study showed no difference. (B) Representative organ histology images showing no indication of tissue toxicity in SPEG injected BALB/c mice after 72 h. (C) The MPI signal acquired from each individual organ collected after 72 h from each cohort is shown. Mann-Whitney **p <0.01.

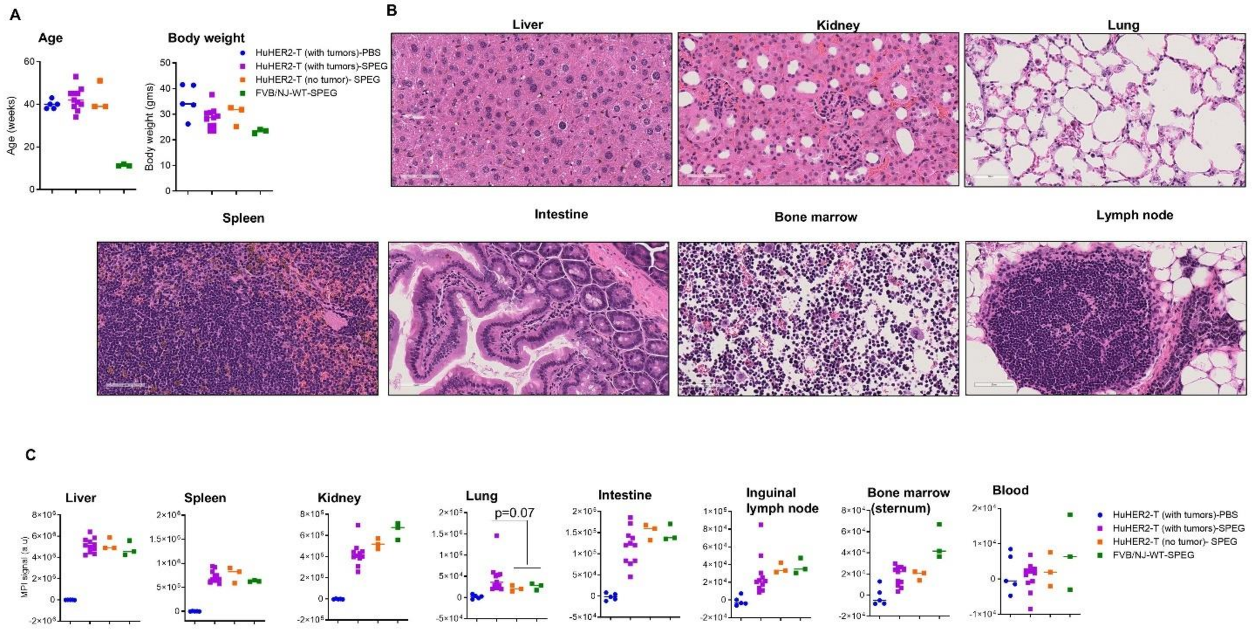

Figure S13: (A) Age and bodyweight of wild type non-tumored FVB/NJ mice, HuHER2 transgenic mice (with and without tumors) used in this study are given here. (B) Representative organ histology images showing no indication of tissue toxicity in SPEG injected 40 weeks old HuHER2 transgenic mice after 72 h. (C) The MPI signal acquired from each individual organ collected after 72 h from each cohort is shown. Mann-Whitney p = 0.07.

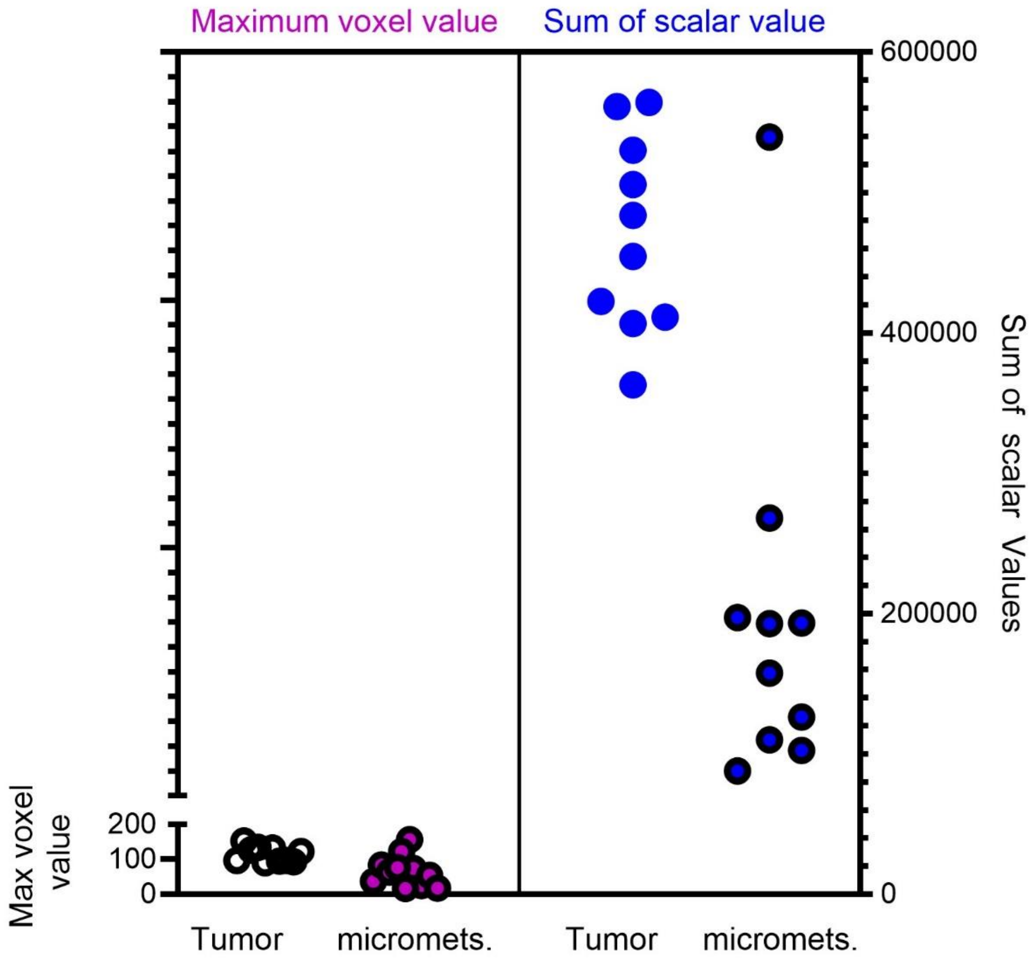

Figure S14: *Ex-vivo* quantitative analysis of primary tumor vs micrometastases in terms of maximum voxel value (highest pixel intensity in an area) and sum of scalar value (total MPI signal in that area) explains the visually similar distinct appearance of high intensity MPI signals in both tumor and micrometastases.

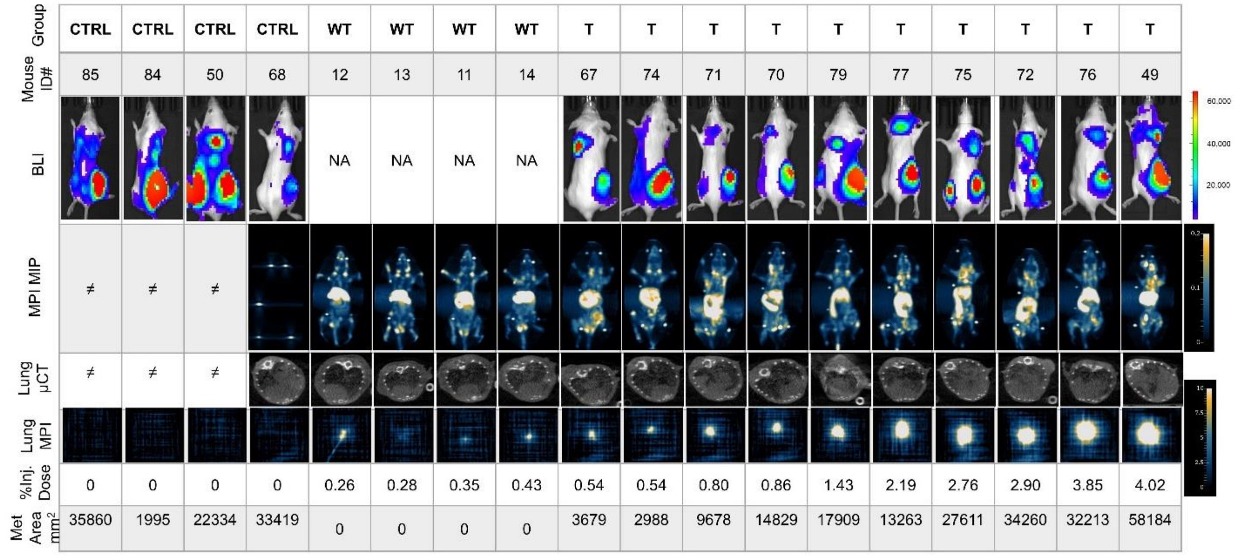

Figure S15: Images acquired from each individual BALB/c mouse with and without 4T1-luc tumor using bioluminescence (BLI), micro-computed tomography (µCT) or MPI (Magnetic Particle Imaging) as described in the Methods. Lung MPI acquired in 2D mode correlates with the measured metastatic area in the lungs. The percentage injected dose in the lung is calculated from the measured 2D MPI signal. (WT – SPEG injected normal non-tumored BALB/c mice, CTRL – control tumored mice that did not receive any nanoparticle injection, T – SPEG injected tumored mice, NA - not applicable, ≠ - no 3D data acquisition only 2D).

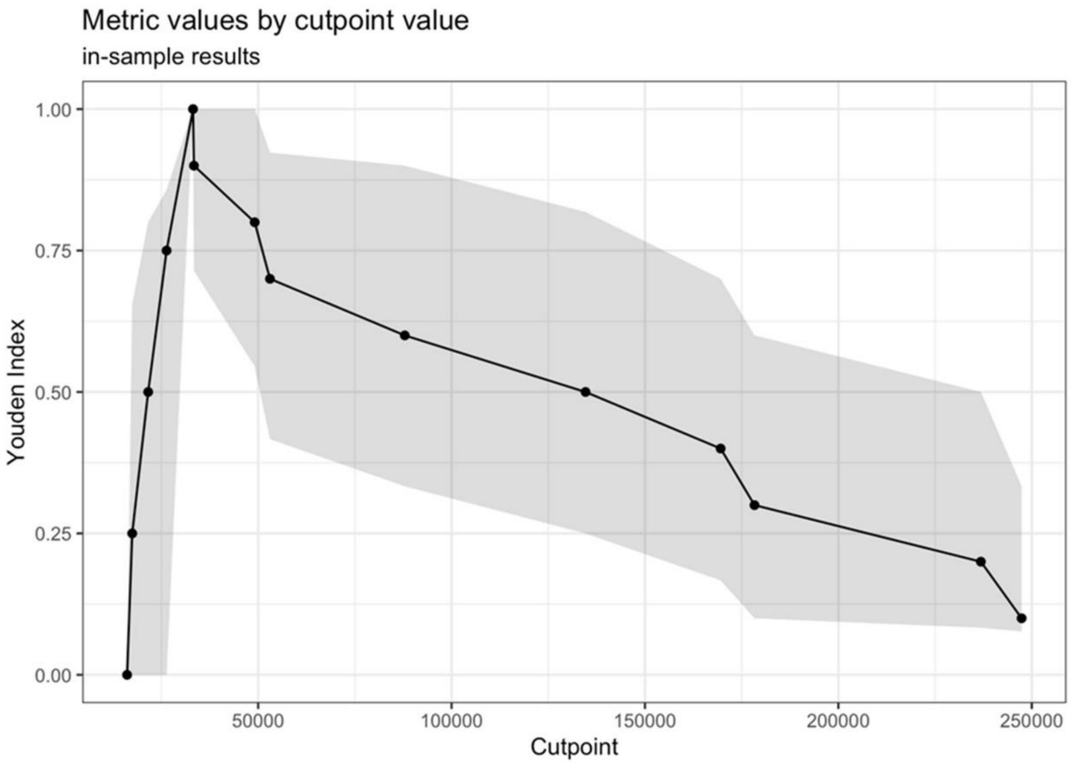

Figure S16: Optimal cutpoint value calculation for 4T1-luc mice.

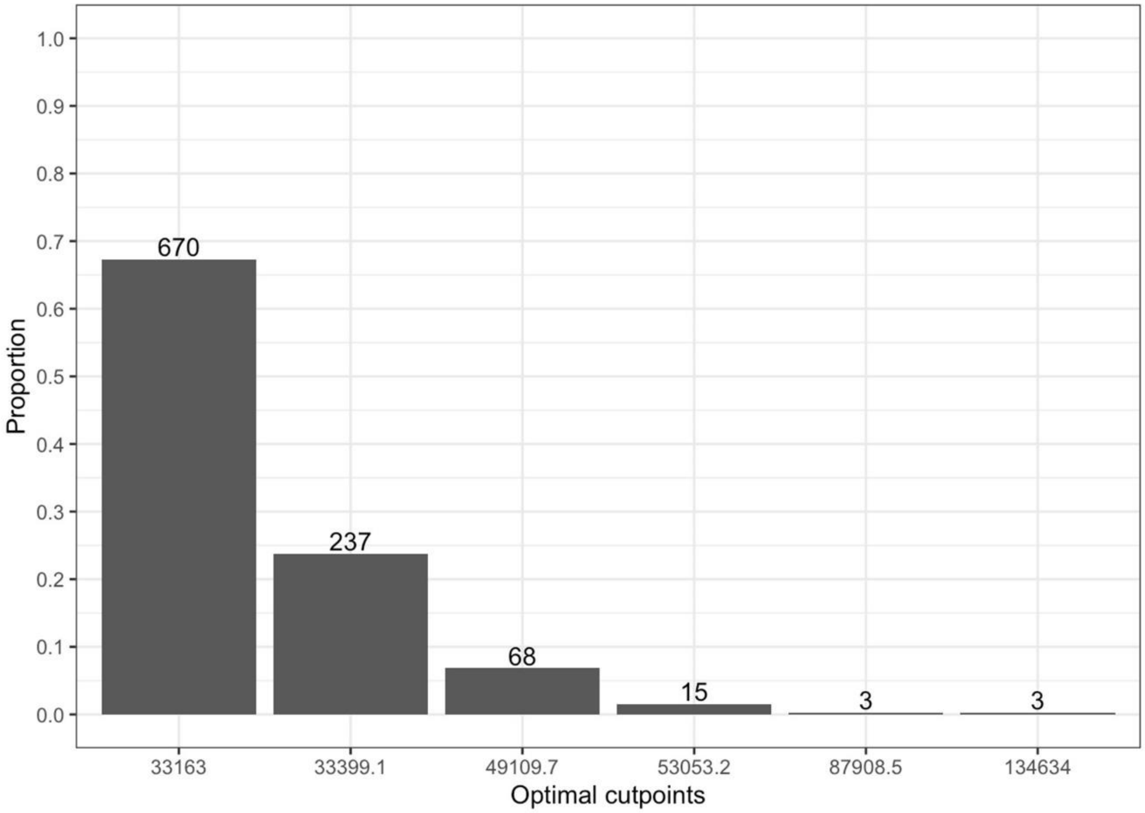

Figure S17: Majority of bootstrap samples showing optimal cutpoint for 4T1-luc mice.

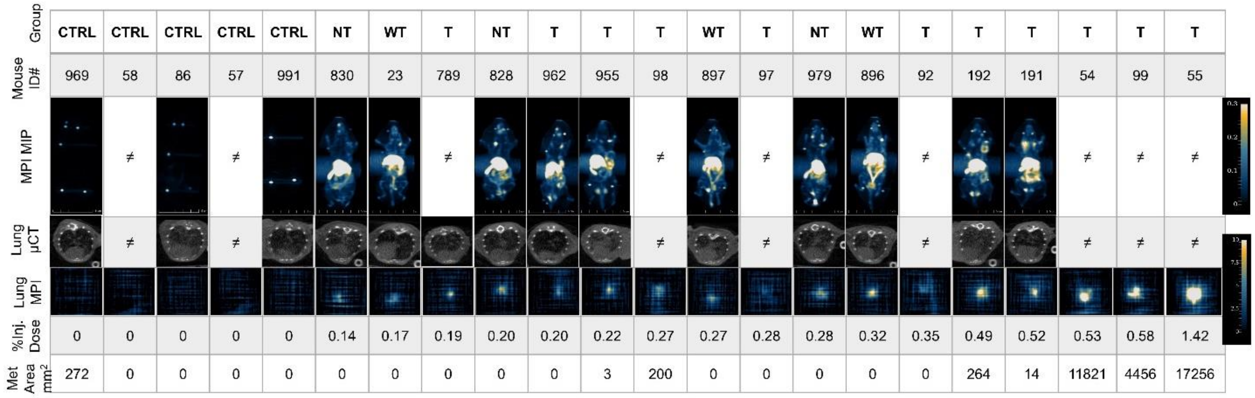

Figure S18: Images acquired from each individual FVB/NJ normal or HuHER2 transgenic mouse with and without tumor using MPI (Magnetic Particle Imaging) or micro-computed tomography (µCT) as described in the Methods. Lung MPI acquired in 2D mode correlates with the measured metastatic area in the lungs. Percentage injected dose in lung is calculated from the measured 2D MPI signal. (WT – SPEG injected normal non-tumored FVB/NJ mice, CTRL – PBS injected tumored HuHER2 transgenic mice that did not receive any nanoparticle injection, T – SPEG injected tumored HuHER2 transgenic mice, NT-SPEG injected age-matched non-tumored HuHER2 transgenic mice, ≠ - no 3D data acquisition only 2D).

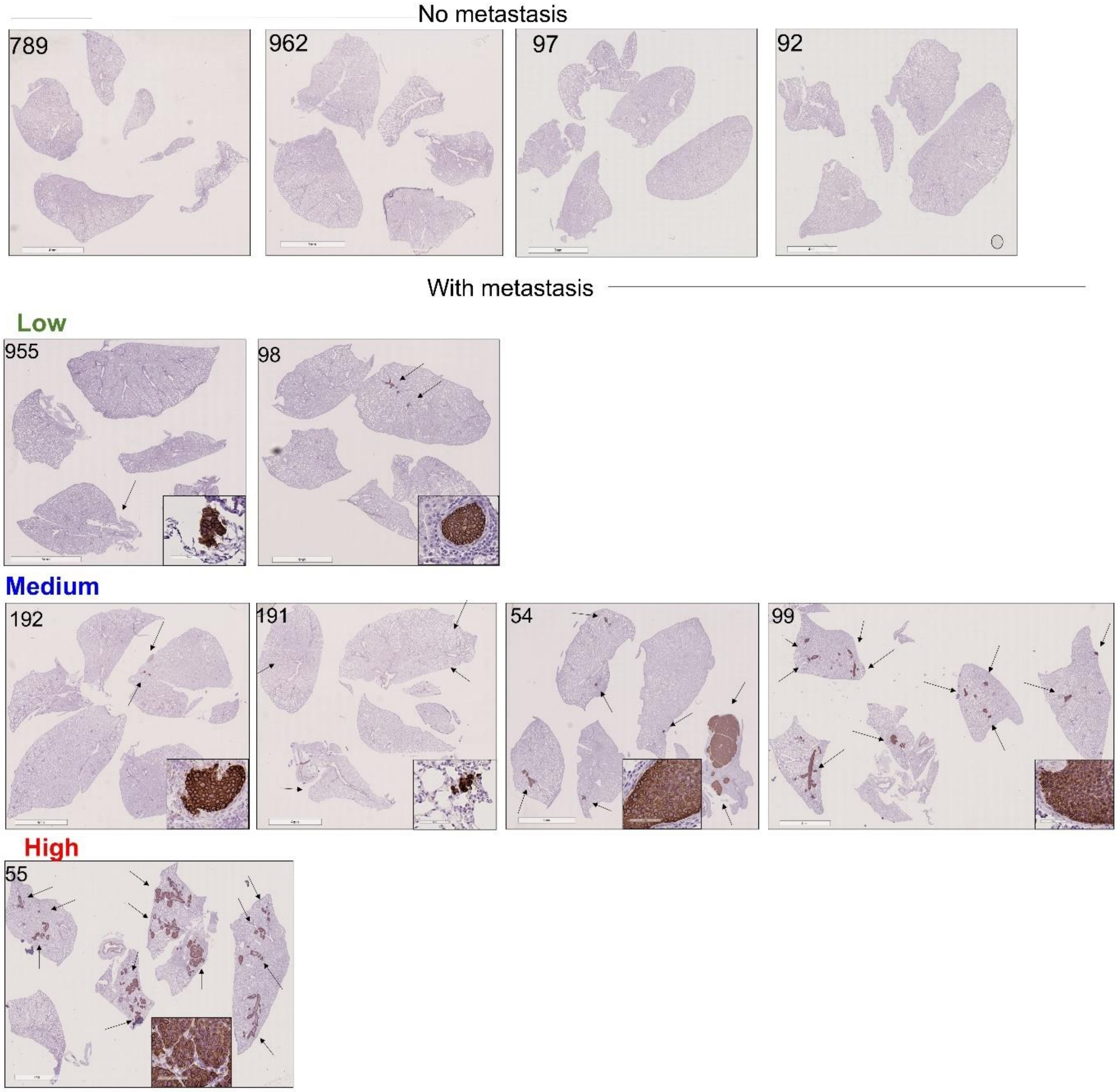

Figure S19: Lungs of individual HuHER2 transgenic mice with tumors injected with SPEG were subjected to HER2 IHC for confirmation of micro and macro metastasis. Individual mouse showed varying number and area of metastatic nodules with 4 out of 11 showed no visible nodules in 5 serial sections of lung tissue (100 µm apart). Those with higher number/area of metastatic nodules (bottom 5) correlated with higher MPI signal.

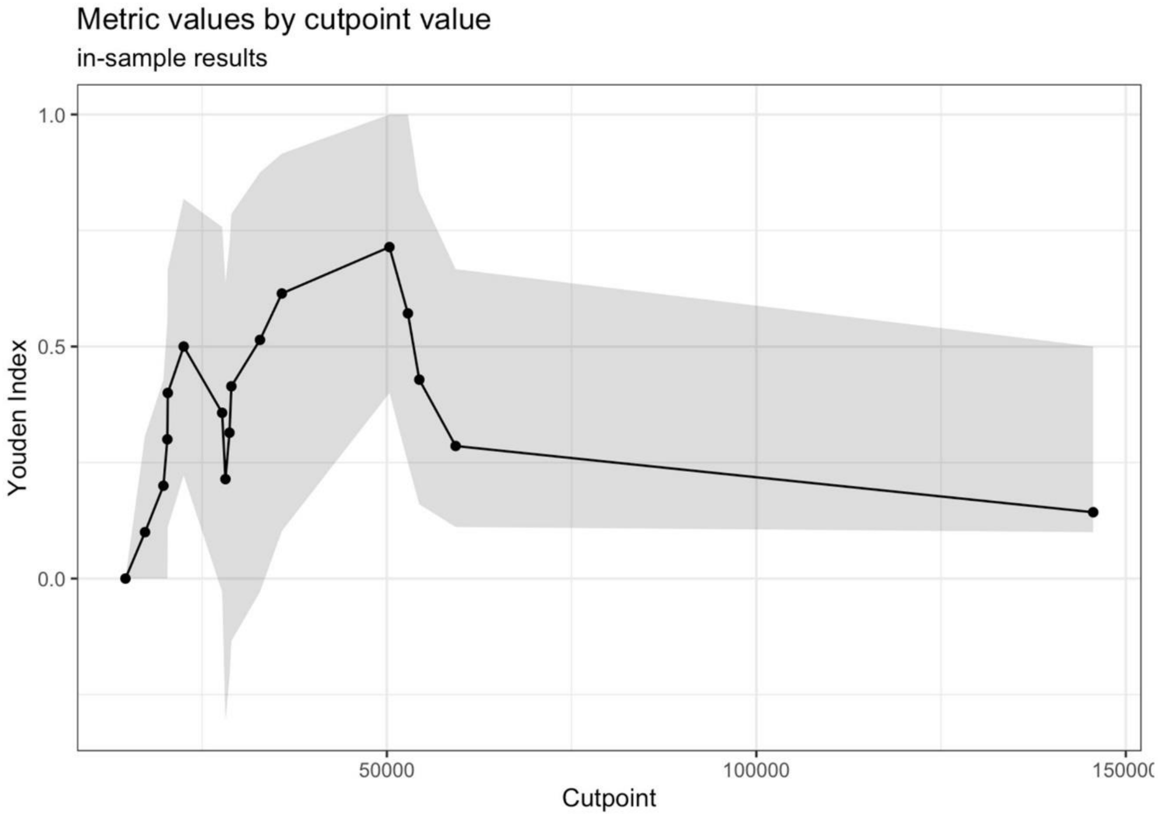

Figure S20: Optimal cutpoint value calculation for huHER2 transgenic mice.

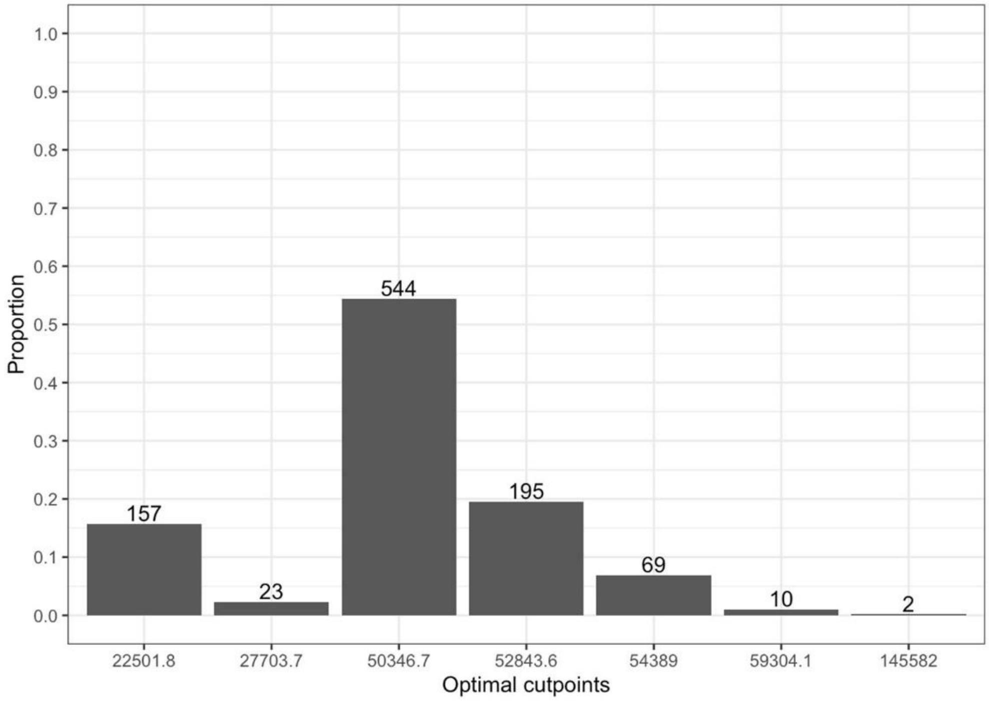

Figure S21: Majority of bootstrap samples showing optimal cutpoint for huHER2 mice.

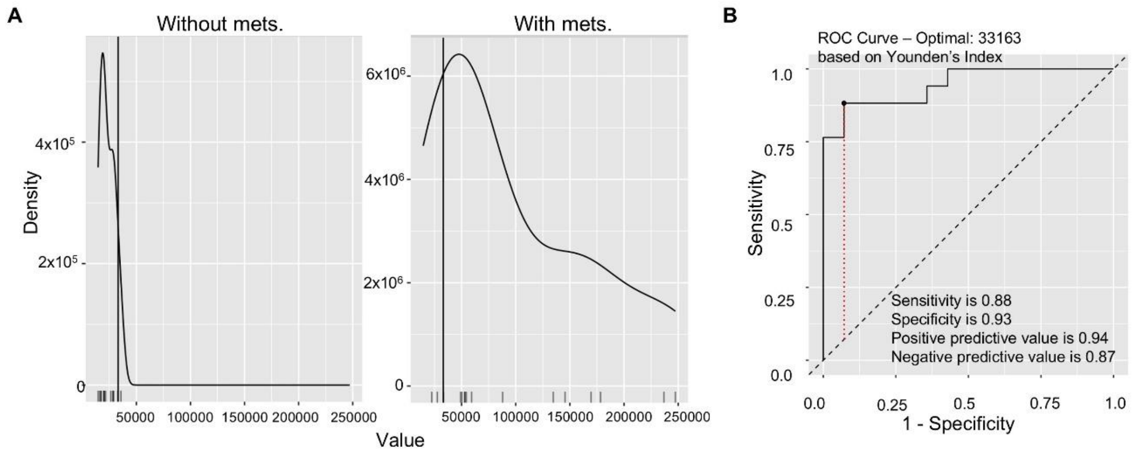

Figure S22: A) Combined statistical analysis of both models shows distinct difference in the distribution of predictor MPI values by outcome for mice without and with metastasis. B) ROC curve for positive and negative prediction shows sensitivity 0.88 and specificity 0.83 for MPI based metastasis detection with calculated optimal cutoff value of 33163.

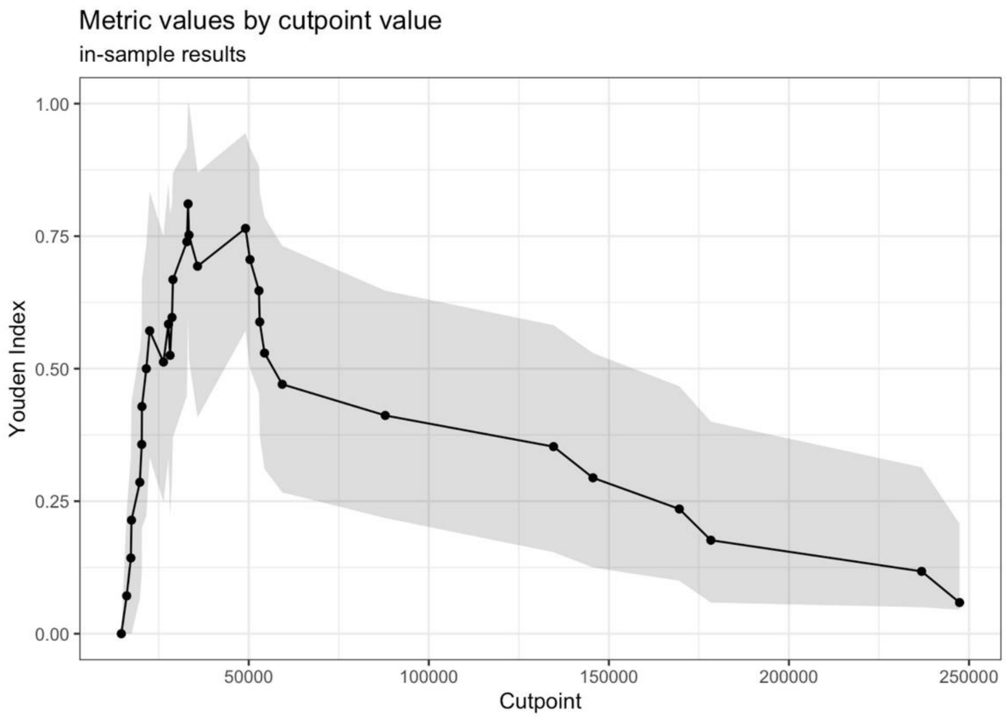

Figure S23: Optimal cutpoint value calculation for all mice combined analysis.

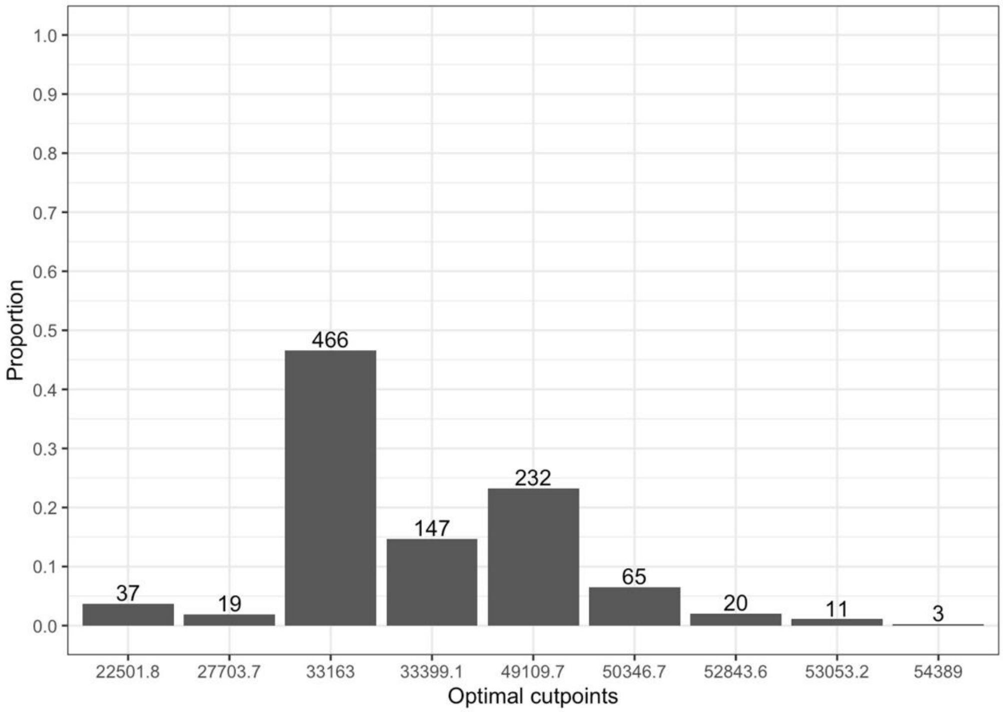

Figure S24: Majority of bootstrap samples showing optimal cutpoint for all mice combined analysis.

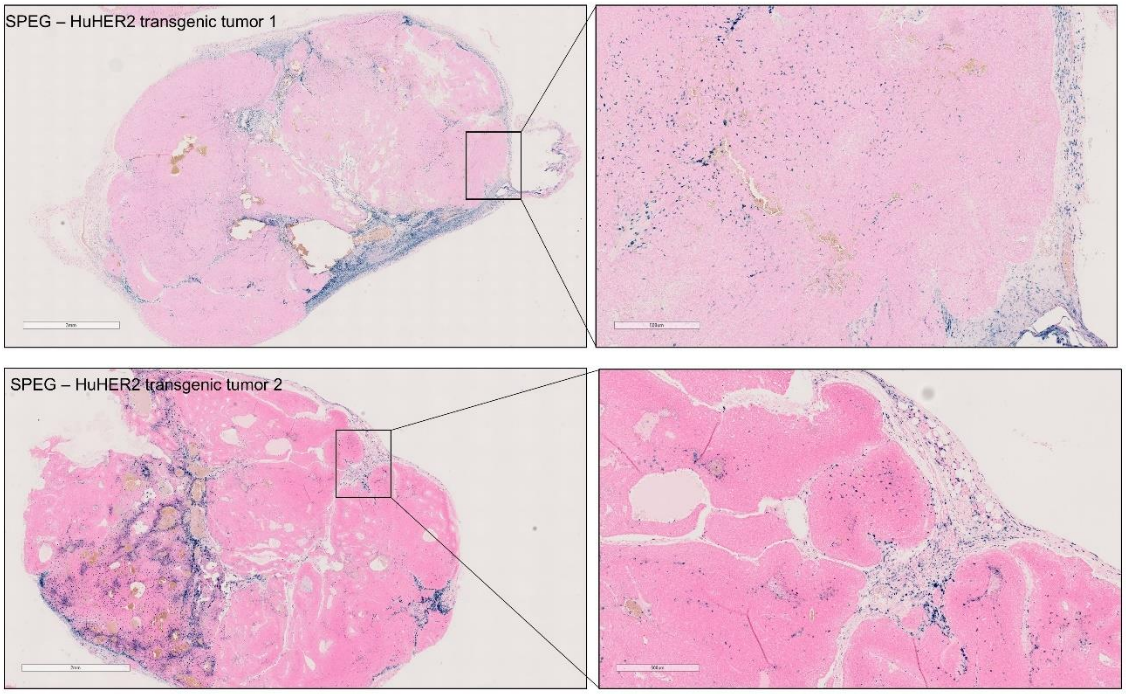

Figure S25: Representative HuHER2 transgenic mice tumors from mice 789 and 192 showing nanoparticles in stromal areas by Prussian blue staining.

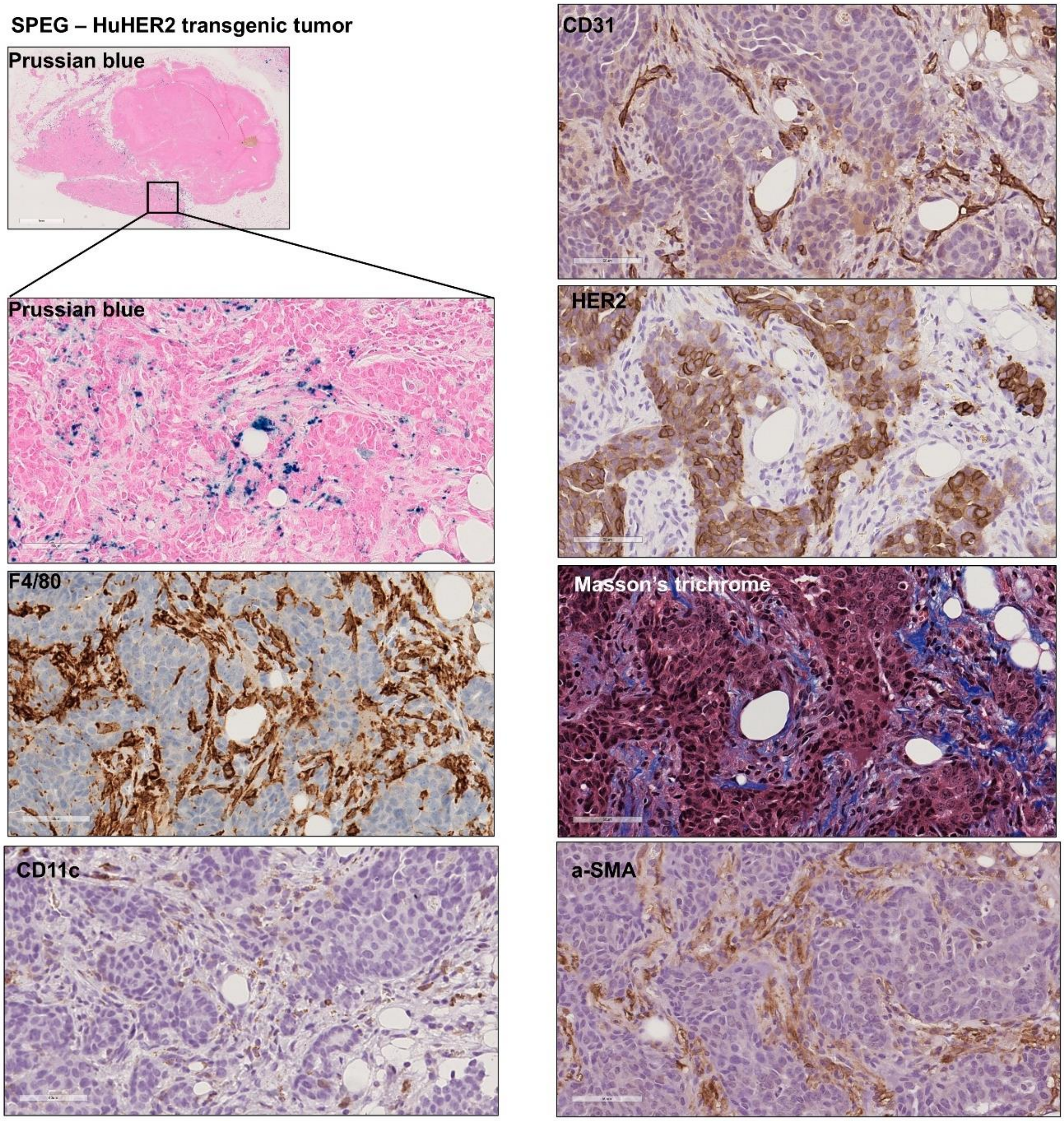

Figure S26: HuHER2 transgenic mice tumors from Mouse 962 were subjected to stromal and immune cell marker immunohistochemistry. This showed nanoparticles are associated with F4/80 macrophages, CD31 endothelial cells, α-SMA fibroblasts, Masson's trichrome collagen than that of HER2+ve tumor cells. Closer evaluation of nanoparticle accumulating areas revealed higher accumulation on normal-tumor boundary regions surrounding the transgenic tumors.

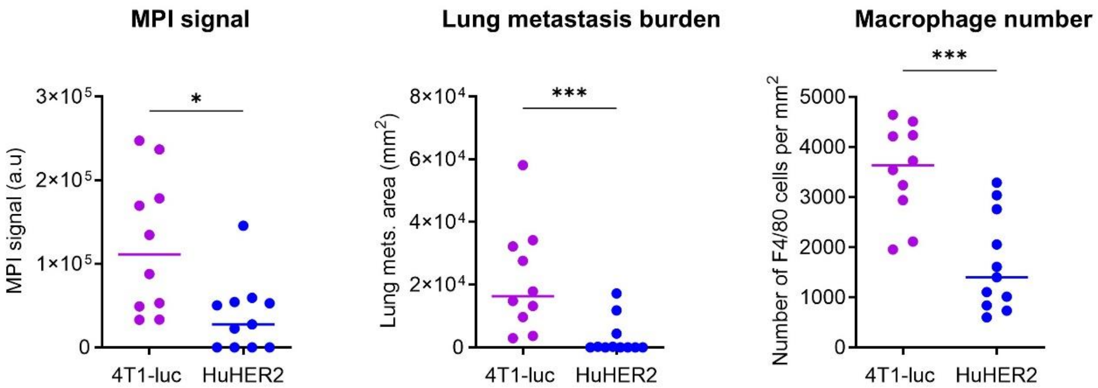

Figure S27: Quantitative comparison of 4T1-luc to HuHER2 lungs shows higher MPI signal in 4T1 lungs than HuHER2 ones due to increased lung metastasis burden and increased accumulation of nanoparticles in higher number of total macrophages in those lungs.

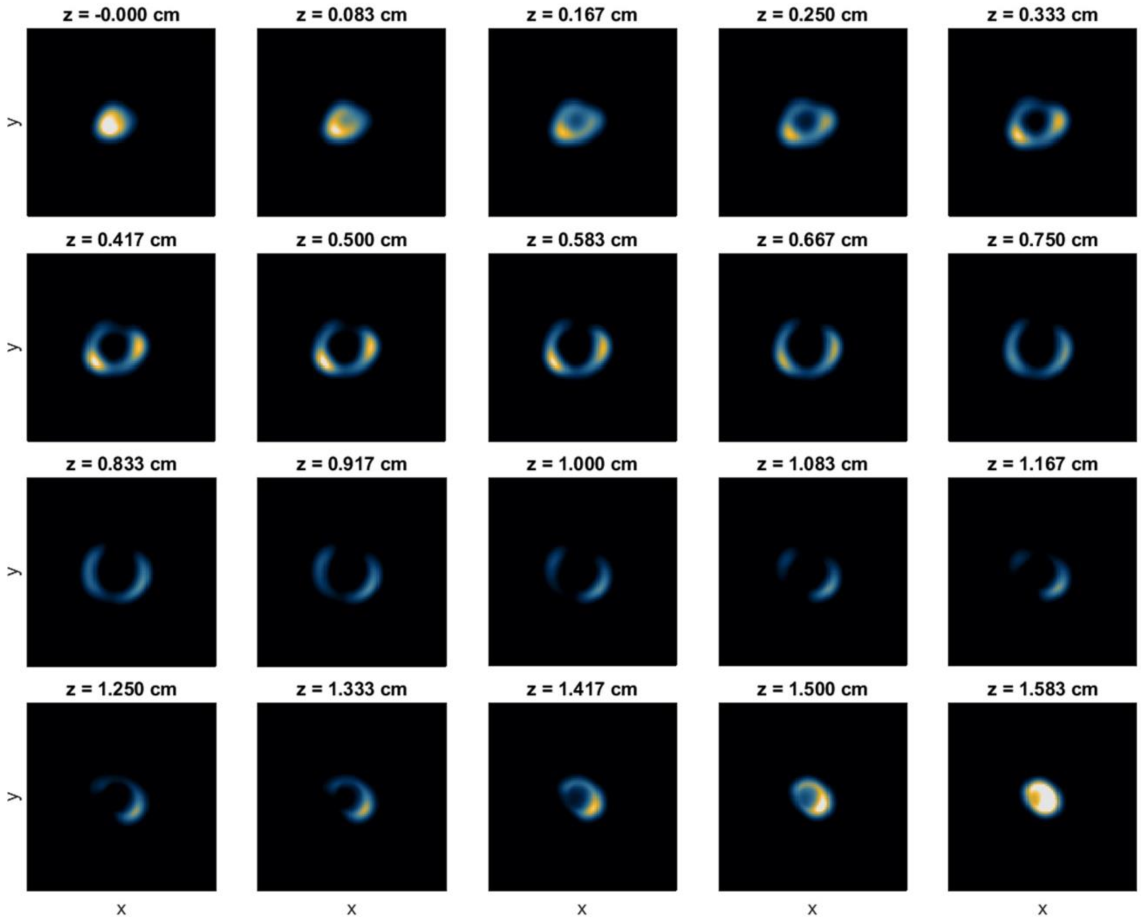

Figure S28: MPI slices of the rim tumor phantom shows the peripheral distribution of 150 μg/mL SPEG throughout the phantom.

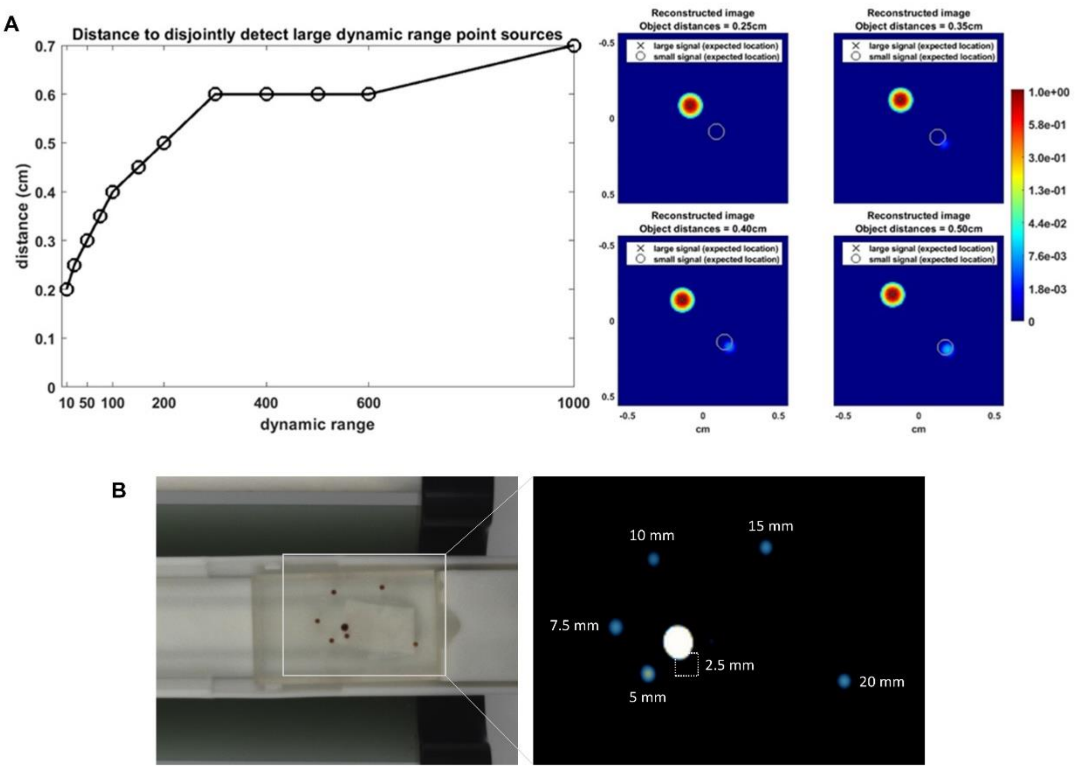

Figure S29 (A) MPI simulation results showing the distance needed to distinguish two point sources at a large dynamic range difference. Simulations are performed for 2D images. (B) Phantom data for shine-through phantom shows two point sources can be resolved with < 5 mm distance with concentration difference of 10x. The central vial contains 300 µg and the six satellite vials contain 30 µg. Imaging was performed in 2D (a single MPI projection).

Table S1.

Physical characteristics of nanoparticles.

Particle type	Coating	Diameter [nm]	PDI	Lot. No:	ZP [mV]
Synomag-PEG (SPEG)	Poly ethylene glycol (PEG 25.000-OMe)	110.9<br>108.2<br>124.7	0.019<br>0.041<br>0.055	38823105-01<br>25724105-01<br>50424105-01	- 8.7<br>- 8.7<br>- 3.6

Table S2.

Actual and predicted positive and negative in 4T1-luc mice.

	Predicted Positive	Predicted Negative
Actual Positive	10	0
Actual Negative	0	4

Table S3.

Actual and predicted positive and negative in huHER2 mice.

	Predicted Positive	Predicted Negative
Actual Positive	5	2
Actual Negative	0	10

Table S4.
Actual and predicted positive and negative in all mice

	Predicted Positive	Predicted Negative
Actual Positive	15	2
Actual Negative	1	13

Movie S1.
Animation of MPI 3D scan of 4T1-luc tumor bearing mouse 72hrs after SPEG injection.

Movie S2.
Animation of MPI 3D scan of huHER2 tumored mouse 72hrs after SPEG injection.

Movie S3.
Animation of MPI 3D scan of huHER2 tumored mouse 72hrs after PBS injection.

Movie S4.
Animation of MPI 3D scan of BALB/c mouse (without any tumor) 72hrs after SPEG injection.

Movie S5.
Animation of MPI 3D scan of FVB/NJ mouse (without any tumor) 72hrs after SPEG injection.

Movie S6.
Animation of MPI 3D scan of age matched huHER2 mouse (without any tumor) 72hrs after SPEG injection.

Movie S7.
Animation of segmented tumor from human glioblastoma used to 3D print tumor phantoms.
